# Gasdermin D Mediated Mitochondrial Metabolism Orchestrate Neurogenesis Through LDHA During Embryonic Development

**DOI:** 10.1002/advs.202402285

**Published:** 2024-07-21

**Authors:** Hongyan Ma, Huiyang Jia, Wenzheng Zou, Fen Ji, Wenwen Wang, Jinyue Zhao, Chenqi Yuan, Jianwei Jiao

**Affiliations:** ^1^ Key Laboratory of Organ Regeneration and Reconstruction, State Key Laboratory of Stem Cell and Reproductive Biology, Institute of Zoology Chinese Academy of Science Beijing 100101 China; ^2^ University of Chinese Academy of Sciences Beijing 100049 China; ^3^ Beijing Institute for Stem Cell and Regenerative Medicine Institute for Stem Cell and Regeneration Chinese Academy of Sciences Beijing 100101 China; ^4^ Co‐Innovation Center of Neuroregeneration Nantong University Nantong 226001 China

**Keywords:** AIFM3, GSDMD, lactate, metabolism, neurogenesis, pyroptosis

## Abstract

Regulatory cell death is an important way to eliminate the DNA damage that accompanies the rapid proliferation of neural stem cells during cortical development, including pyroptosis, apoptosis, and so on. Here, the study reports that the absence of GSDMD‐mediated pyroptosis results in defective DNA damage sensor pathways accompanied by aberrant neurogenesis and autism‐like behaviors in adult mice. Furthermore, GSDMD is involved in organizing the mitochondrial electron transport chain by regulating the AMPK/PGC‐1α pathway to target Aifm3. This process promotes a switch from oxidative phosphorylation to glycolysis. The perturbation of metabolic homeostasis in neural progenitor cells increases lactate production which acts as a signaling molecule to regulate the p38MAPK pathway. And activates NF‐𝜿B transcription to disrupt cortex development. This abnormal proliferation of neural progenitor cells can be rescued by inhibiting glycolysis and lactate production. Taken together, the study proposes a metabolic axis regulated by GSDMD that links pyroptosis with metabolic reprogramming. It provides a flexible perspective for the treatment of neurological disorders caused by genotoxic stress and neurodevelopmental disorders such as autism.

## Graphical abstract

Pyroptosis during cortical development occurs in response to high levels of replicative stress and contributes to the elimination of damaged neural progenitor cells. GSDMD deficiency causes DNA accumulation and reprograms the metabolic profile of neural stem cells by affecting the way mitochondria metabolize energy during embryonic development. Also associates with autism‐like behavior in adult mice after birth.

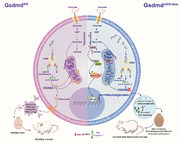

## 1 Introduction

Brain cortical development is dynamically regulated primarily through coordinated neurogenesis and migration processes.^[^ ^1^, ^2^ ^]^ Cortical neurogenesis occurs in the early stages of embryonic development. During this process, neural progenitor cells (NPCs) located in the ventricular zone (VZ) self‐renew through symmetric divisions, and asymmetric divisions of progenitors generate neurons and glial cells.^[^ ^3^, ^4^ ^]^ As corticogenesis progresses, newly generated postmitotic neurons travel to the cortical plate, creating six layers of the cortex.^[^ ^5^, ^6^ ^]^ These layers are meticulously regulated by genes and external signaling. Any abnormal stimuli or disturbances can result in neurodevelopmental disorders.^[^ ^5^, ^6^ ^]^

Cerebral cortical development is characterized by rapid and massive proliferation and differentiation of neural stem cells, followed by extensive cell death, in which over half of the newborn cells are pruned. A large amount of DNA damage, mitochondrial stress, and cellular debris are generated during development^[^ ^7^, ^8^ ^]^ all of which may activate endogenous immune signaling. Regulatory cell death (RCD) is an evolutionarily conserved factor in the development of the nervous system and includes various types such as apoptosis, pyroptosis, necrosis, and ferroptosis, each with its molecular mechanisms. Before this, death during neurogenesis was thought to be solely through apoptosis; however, recent years of in‐depth study, have revealed the need to revisit the modes of cell death, including pyroptosis. However, the regulatory mechanisms and significance of RCD in cortical development are not fully understood.^[^ ^7^, ^8^ ^]^ Numerous studies have shown that pro‐apoptotic gene depletion leads to various central nervous system (CNS) developmental disorders and mortality in both mice and *Drosophila*.^[^ ^7^, ^8^ ^]^ However, the effects of other cell death defects on cerebral cortex formation remain unclear. In recent years, more attention has been paid to the role of pyroptosis in cortical development. Pyroptosis is an inflammatory caspase‐1‐dependent mechanism that involves the activation of interleukin‐1β (IL‐1β) and interleukin‐18 (IL‐18) by caspase‐1, along with the targeting of gasdermin D (GSDMD), a pore‐forming protein. Caspase‐1 cleaves gasdermin D and releases its N‐terminal domain. The domain then migrates to the membrane and forms pores, leading to cell swelling and rupture, a process known as pyroptosis.^[^ ^9^ ^]^ Most studies have focused on pyroptosis activated by different inflammatory diseases;^[^ ^9^ ^]^ for example, in Alzheimer's disease, neuronal pyroptosis in response to stimulation is NLRP1‐dependent and pyroptosis by the activation of inflammasome components NLRC4 and AIM2 in the brain after ischemic stroke.^[^ ^10^ ^]^ During typical cerebral cortex development, pyroptosis responds to endogenous immune signals like DNA damage mitochondrial stress, and other signals, these signals activate inflammatory AIM2,^[^ ^11^ ^]^ which is a component of the enzyme caspase‐1 and the adaptor protein ASC.^[^ ^12^ ^]^ Defects in this DNA damage sensor result in reduced neuronal cell death and anxiety‐related behaviors in mice.^[^ ^13^ ^]^ Gasdermin D is critical in several cell types, including intestinal epithelial cells and neutrophils.^[^ ^14^, ^15^ ^]^ The GSDMD‐NT binding in the mitochondrial membrane, resulting in severe morphological damage, disruption of electron transport, induction of reactive oxygen species, among other effects. Mitochondria provide adenosine triphosphate (ATP) for growth, and regeneration of the nervous system, and bear the burden of maintaining calcium homeostasis. In the central nervous system, dysfunctional mitochondria can a variety of neurological disorders such as autism, depression, or Parkinson's disease.^[^ ^16^ ^]^ However, its function and mechanisms in cortical development remain unknown.

In this study, we observed pyroptosis during the initial stages of cerebral cortical development. The absence of GSDMD resulted in reduced cell death and excessive accumulation of DNA damage in neural stem cells throughout development, whereas in embryonic development, the lack of GSDMD promoted neural progenitor cell proliferation and inhibited neuronal differentiation and these abnormalities led to autism‐like behaviors in adult mice. In subsequent mechanistic analyses, GSDMD is involved in the organization of the mitochondrial electron transport chain by regulating the AMPK/PGC‐1α pathway to target Aifm3. This process promotes a switch from oxidative phosphorylation (OXPHOS) to glycolysis. The absence of GSDMD‐mediated pyroptosis results in mitochondria dysfunction. The perturbation of metabolic homeostasis in neural progenitor cells increases lactate production which acts as a signaling molecule to regulate the p38MAPK/Rac1 pathway. And activates NF‐𝜿B transcription to disrupt cortex development.

Overall, this study demonstrated a previously undiscovered role of GSDMD in the development of the cerebral cortex and revealed a correlation between pyroptosis and cellular metabolism. Here, we propose a GSDMD‐regulated metabolic axis specific for neurogenesis. This metabolic axis is genetically responsible for metabolic reprogramming. It may help open new therapeutic perspectives for neurological disorders caused by innate immune injury and neurodevelopmental disorders such as autism.

## 2 Results

### 2.1 GSDMD Bridges Extrinsic and Intrinsic Pyroptosis of NPCs

**Figure 1:**
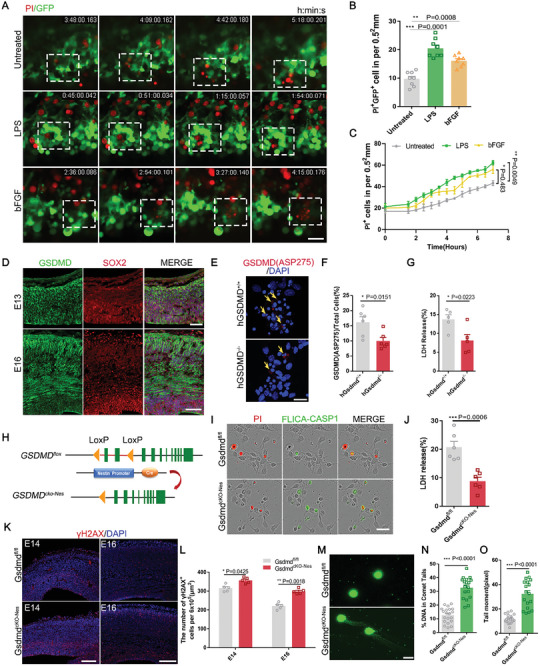
Gasdermin D mediated pyroptosis in vivo and in vitro and correlated with the accumulation of DNA damage. A) GFP‐expressing plasmid was electroporated into the E13 cerebral cortices, and the brain vibratome sections were treated LPS (1 ug mL^−1^) or bFGF (50, 500 ng mL^−1^) and PI dye in the medium. Shown are representative time‐lapse cell images. Scale bar, 50 µm. B)Statistics show that the number of PI^+^GFP^+^ cells. *n* = 8 field of view of 3 individual experiments. C) Statistics show the number of untreated and treated LPS or bFGF PI^+^ cells. D) Confocal immunofluorescence image of GSDMD and SOX2 at E13 and E16. GSDMD is co‐labeled with Sox2 in neural progenitor cells in vivo. Scale bars, E13, 40 µm, E16, 50 µm. E) WT and GSDMD‐KO human NPCs cells were fixed and costained with GSDMD(ASP275) antibodies. Scale bar, 20 µm. F) Statistics show the number of GSDMD (ASP275)^+^cells in GSDMD^+/+^ and GSDMD^−/−^ groups. *n* = 6 independent experiments. G) Lactate dehydrogenase (LDH) release was measured in supernatant derived from human NPCs with control and h*GSDMD*‐shRNA lentivirus‐infected. *n* = 6 independent experiments. H) Schematic of the *GSDMD* conditional knockout (cKO) mice construction strategy. The *GSDMD* gene was knocked out by NPCs‐specific Cre recombinase splicing. I) NPCs were isolated from *Gsdmd ^fl/fl^* mice and *Gsdmd* ^cKO‐Nes^ mice and cultured in vitro for 24 h, and cells were stained with FLICA‐CASP1 for 1 h and Propidium iodide. The images were visualized using microscopy. Scale bar, 50 µm. J) LDH release was measured in supernatant derived from NPCs of *Gsdmd ^fl/fl^* mice and *Gsdmd^cKO‐Nes^* mice. *n* = 6 independent experiments. K) Images of cerebral cortex sections of E14, and E16 labeled for 𝛾H2A.X and DAPI. Left: scale bar, 50 µm. Right: scale bar, 50 µm. L)The bar graph shows the number of 𝛾H2A.X^+^ cells in the VZ/SVZ per 6 × 10^5^ µm^2^. *n* = 5 independent experiments. (M) DNA damage was evaluated in the E13 NPCs of *Gsdmd^fl/f^* mice and *Gsdmd^cKO‐Nes^* mice by comet assay. Representative images of single‐cell electrophoresis gels from 3 independent experiments with similar results. N) Quantification of the percentage of DNA in the tail (*Gsdmd^fl/fl^ n* = 20, *Gsdmd^cKO‐Nes^*, *n* = 18, from 3 independent experiments). O)Quantification of the comet tail moment from *Gsdmd^fl/fl^* mice and *Gsdmd^cKO‐Nes^* mice. (*Gsdmd^fl/fl^ n* = 20, *Gsdmd^cKO‐Nes^*, *n* = 18 from 3 independent experiments). Error bars represent means ± SEMs; 2‐tailed unpaired *t*‐test; one‐way ANOVA with Dunnett's multiple‐comparison correction. ^*^ *p* < 0.05, ^**^ *p* < 0.01, ^***^ *p* < 0.001; n.s., not significant.

Various regulatory cell death pathways, such as GSDMD‐mediated pyroptosis are required for healthy brain development. We first observed that cells exhibiting typical pyroptosis progressed with the development of characteristic swelling bubbles and ruptured in the neural progenitor cells when cultured in vitro (Figure S1A, Supporting Information). In addition, we observed various cell death pathways in human neural precursor cells (NPCs), including apoptosis and pyroptosis (Figure S1C, Supporting Information). To dynamically observe the specific phenomenon that cell membranes bubble up and burst when pyroptosis occurs in vivo, we electroporated a GFP plasmid into the VZ/SVZ region of the brain of E13 mice. and then dissected the living brain tissues by vibratory sectioning after 24 h. And we observed that in GFP cells was observed, and green fragments emerged, red dye gradually entered the cells from the ruptured holes. Since the red dye gradually entered the cells from the ruptured holes and finally became red cell fragments, this change is the occurrence of pyroptosis cells (**Figure** **1**A). In addition to observing the onset of pyroptosis in vitro using live brain slices, we further distinguished between pyroptosis and apoptosis in vivo by co‐labeling with GSDMD‐NT. We found that Annexin V hardly co‐localized with GSDMD‐N but most of the propidium iodide (PI) did by staining the cortex of mice at developmental stages E13 and E15 for co‐localization of GSDMD‐N with PI and annexin V, respectively (Figure S1C, Supporting Information).

Most of the known pyroptosis occurs in an inflammatory environment. Therefore, we used LPS to simulate the pathological environment of bacterial invasion. LPS is able to cleave GSDMD after recognizing caspase‐4/5/11, an immune receptor for inflammatory signals.^[^ ^17^, ^18^ ^]^ And  basic fibroblast growth factor (bFGF), a widely known trophic factor for neural stem cell proliferation, is a key factor regulating neurogenesis in the central nervous system, which can emulate the proliferative environment in vivo.^[^ ^19^, ^20^ ^]^ Pyroptosis occurred less frequently during normal development and over a longer period than stimulation with LPS or bFGF growth factors (Figure 1B,C). We hypothesized that mitochondrial stress and cellular debris would result from the high levels of replication stress that arise during the peak of cortical development, which may stimulate immune signaling and increase pyroptosis. To test our hypothesis, we cultured primary neural progenitor cells in vitro under long‐term observation and found that the uptake of PI increased significantly with increasing concentrations of bFGF and LPS (Figure S2A,B, Supporting Information).

Along with higher protein levels of cleaved GSDMD and increased IL‐Iβ secretion (Figure S2D,E, Supporting Information), we observed elevated lactate dehydrogenase (LDH) levels in the supernatant (Figure S2C, Supporting Information). The above experiments indicated that pyroptosis is closely related to developmental processes; thus, we investigated the role of GSDMD as a pyroptosis executor in this association. First, we examined changes in GSDMD expression in the brain during different developmental periods. We found that its expression gradually increased with development (Figure S2G, Supporting Information), and in the initial stage of development, the expression was mainly in the VZ/SVZ region, which was co‐labeled with Sox2. With the developmental process, it also became expressed in the cortex (Figure 1D). To confirm the in vitro expression of GSDMD, primary neural progenitor cells isolated from E13 mouse brains were analyzed. Immunostaining revealed that GSDMD was co‐labeled with Sox, Pax6, and Tuj1, indicating that GSDMD was expressed in both neural progenitor cells and neurons in the developing cortex (Figure S2F, Supporting Information). To confirm the presence of pyroptosis in human NPC proliferation, we constructed a human *GSDMD* knockout cell. After gene identification, knockdown efficiency was measured at the protein level, and the cell lines with the highest knockdown efficiency were selected for subsequent experiments (Figure S2H,I. Supporting Information). We observed reduced cleaved GSDMD and cell lysis in human NPCs after GSDMD knockout according to immunofluorescence staining of the amino acid ASP275 at the cleavage end of GSDMD (Figure 1E,F). we also observed elevated lactate dehydrogenase (LDH) levels in the human NPC cell supernatant (Figure 1G). The above experiments explored the phenotype of pyroptosis in vivo and in vitro and verified pyroptosis as part of regulatory cell death in response to immune injury from high levels of replication at the peak of cerebral cortical development. The expression changes and positional relationships of GSDMD led us to speculate that GSDMD may have an undiscovered and unique function in neurogenesis during brain development.

### 2.2 Disruption of Pyroptosis Impairs DNA Damage Clearance and AIM2 Inflammasome in Neural Progenitor Cells

High levels of replicative stress during development can result in immune damage, inflammation, and GSDMD‐mediated pyroptosis. We bred Nestin‐Cre mice with *Gsdmd^fl/fl^* mice to generate GSDMD conditional knockout (*Gsdmd^cKO‐Nes^*) mice (Figure 1H), and their efficiency was examined at both the protein and RNA levels (Figure S3C–E, Supporting Information). We also assayed changes in GSDMD expression in the cerebral cortex (Figure S3A,B, Supporting Information). To further validate GSDMD‐driven pyroptosis, we isolated primary neural progenitor cells from *Gsdmd^fl/fl^* and *Gsdmd^cKO‐Nes^* E13 mice cultured in vitro and double‐stained them for active caspase‐1 and PI. We found that PI‐positivity was significantly decreased and active caspase1 was increased in *Gsdmd^cKO‐Nes^* (Figure 1I; Figure S3F,G, Supporting Information). We detected a substantial reduction in cell death in neural progenitor cells (Figure 1J). The same phenomenon was observed in neural progenitor cells that used disulfiram, which antagonizes the GSDMD N‐terminus to inhibit pore formation (Figure S3H,I, Supporting Information). Our results suggest that pyroptosis in NPCs is dependent on oligomerization of the N‐terminal GSDMD.

During brain development, high levels of replication stress and cell death generate various injury or danger signals such as mitochondrial stress, and DNA damage, all of which can activate inflammatory signaling. The absence of GSDMD results in impaired clearance of cells with DNA damage, which accumulated in the VZ/SVZ region (Figure 1K,L). We examined DNA damage in neural progenitor cells using the comet assay and cells isolated from *Gsdmd^fl/fl^* and *Gsdmd^cKO‐Nes^* mice, DNA content, and comet tail moment were both significantly higher in the GSDMD‐depleted mice (Figure 1M–O).

In previous experiments, we found that increasing bFGF concentration increased pyroptosis when culturing NSCs in vitro, so we further investigated whether DNA damage is predominantly present in rapidly proliferating NPCs, which activates AIM2 inflammation. The immunostaining and western blot analyses showed that the phosphorylation level of histone γH2AX correlates with the concentration of bFGF (Figure S3J–M, Supporting Information). This also confirmed that high replication stress can increase DNA damage. Immunofluorescence staining showed that the phosphorylation level of histone γH2AX was significantly increased in the *GSDMD* knockout groups (Figure S3N,O, Supporting Information).

In GSDMD deficient mice, the accumulation of DNA damage activated more receptor AIM2 inflammasomes (Figure S4A,C, Supporting Information), accompanied by a significant increase in cleaved caspase‐1 (Figure S4B,D, Supporting Information), we have observed higher levels of ASC speck formation in the brain of *Gsdmd^cKO‐Nes^* mice (Figure S4E,F, Supporting Information), and the same changes were found at the protein level in the mouse cerebral cortex (Figure S4G,H, Supporting Information). DNA damage is a major cause of induced cell death during development under physiological conditions, does deletion of the DNA receptor AIM2 function in the same way for neurogenesis. Therefore, we constructed a knockdown plasmid of AIM2, and analyzed the fluorescence distribution and immunofluorescence staining by IUE of the AIM2‐shRNA plasmid at E13. Knockdown of AIM2 increased the number of DNA‐damaged cells in the VZ/SVZ region of the brain (Figure S4I–K, Supporting Information). But the distribution of GFP‐positive cells and the analysis of co‐localization with SOX2‐positive cells showed that AIM2 knockdown did not affect the neurogenesis process (Figure S4L,M, Supporting Information), but affected the efficiency of intracellular DNA‐damaged cell clearance.

We have found that the AIM2 inflammasome signaling pathway is involved in the DNA damage‐induced pyroptosis of cortical neural progenitor cells during development. From the above experiments, we concluded that GSDMD participates in the clearance of DNA‐damaged cells in the developing cortex and that the absence of GSDMD disrupts the pyroptosis pathway, leading to increased levels of DNA‐damaged cells and their accumulation in the brain.

### 2.3 GSDMD Deletion Disrupts Early Neurogenesis

**Figure 2:**
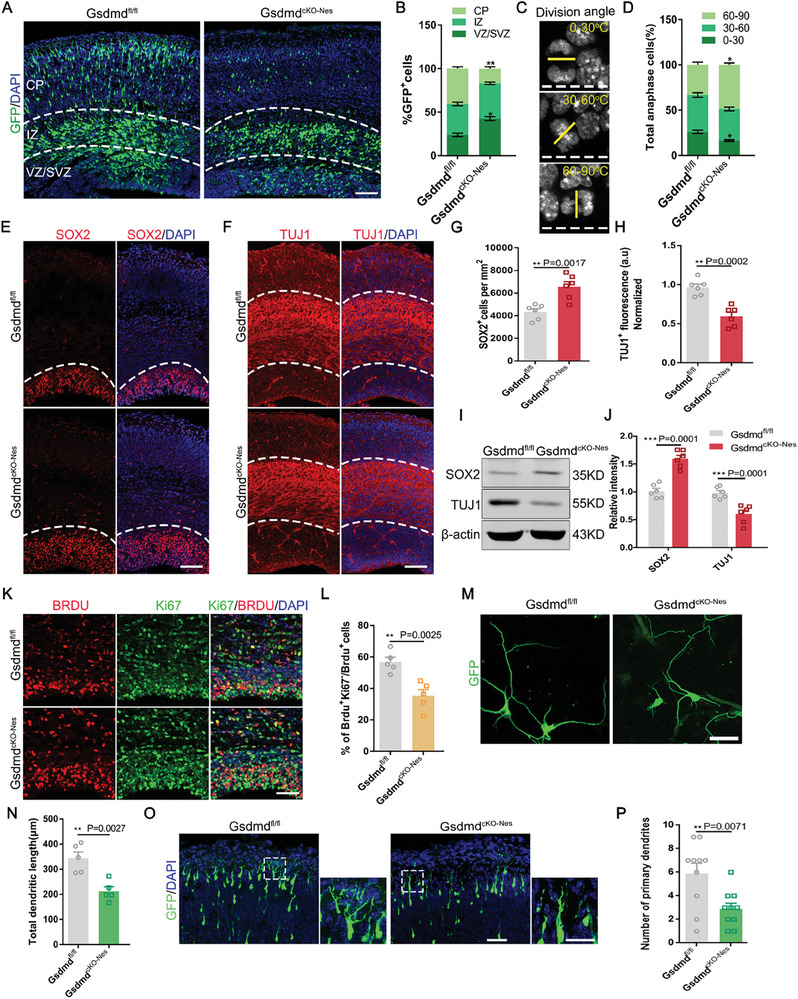
Gasdermin D deletion in the brain impairs neurogenesis. A) Abnormal cell distribution is observed in GSDMD ablated neocortex. The GFP plasmid was electroporated into the E13 mouse brains of *Gsdmd^fl/fl^* mice and *Gsdmd^cKO‐Nes^* mice, and the mice were sacrificed at E16. Scale bars, 50 µm. B) Graphs of the percentage of GFP‐positive cells in the VZ/SVZ, IZ, and CP. *n* = 6 independent experiments. C) Division angle measurements of NPCs in E15 coronal sections. Horizontal (0−30 °C), intermediate (30−60 °C), and vertical (60−90 °C) cleavage angles (yellow line) relative to the apical surface of the lateral vertical. Chromatin is visualized by DAPI. D) Division angle measurements of anaphase cells in *Gsdmd^fl/fl^* mice and *Gsdmd^cKO‐Nes^*. (*Gsdmd^fl/fl^ n* = 16, *Gsdmd^cKO‐Ne^ n* = 18, from 3 independent experiments). E)Brain sections of *Gsdmd^fl/fl^* mice and *Gsdmd^cKO‐Nes^* mice at E16 were immunostained with SOX2 and DAPI. Scale bar,100 µm. F) Brain sections of *Gsdmd^fl/fl^* mice and *Gsdmd^cKO‐Nes^* mice at E16 were immunostained with TUJ1 and DAPI. Scale bar,100 µm. G) Quantitative analyses of the SOX2^+^ cells at the E16 cerebral cortex. *n* = 6 independent experiments. H) Quantification of the Tuj1 fluorescence intensity in *Gsdmd^fl/fl^* mice and *Gsdmd^cKO‐Nes^* mice. a.u., arbitrary units. *n* = 6 independent experiments. I) Western blot analysis of SOX2, TUJ1 protein levels in E16 *Gsdmd^fl/fl^* mice and *Gsdmd^cKO‐Nes^* mice. J) The bar graph shows the normalized densitometry of SOX2, TUJ1. *n* = 6 independent experiments. K) Cell‐cycle exit was decreased in *Gsdmd^cKO‐Nes^* mice. *Gsdmd^fl/fl^* mice and *Gsdmd^cKO‐Nes^* mice were administered BrdU (100 mg k^−1^g) for 24 h and were euthanized at E16. E16 brain sections were stained with anti‐BrdU and anti‐Ki67. Scale bars, 50 µm. L) Percentage of cell‐cycle exit (BrdU^+^Ki67^‐^/BrdU^+^) in *Gsdmd^fl/fl^* mice and *Gsdmd^cKO‐Nes^* mice. *n* = 5 independent experiments. M) Representative images of neurons after 4 days of culture in vitro. The GFP‐expressing plasmid was electroporated into the E13 cerebral cortices of *Gsdmd^fl/fl^* mice and *Gsdmd^cKO‐Nes^* mice to mark neural progenitor cells. After 24 hours, the GFP‐positive cells were isolated and cultured in a differentiation medium. Scale bar, 20 µm. N) The graph shows that the total dendritic length of neurons is decreased when *GSDMD* is deleted. *n* = 5 independent experiments. O) IUE was performed in the mice at E13.5 and analyzed at P0. GFP‐positive cells show that GSDMD knockout has abnormal leading processes compared to the WT. Scale bar, 50 µm. P) Quantification of primary dendritic numbers in *Gsdmd^fl/fl^* mice and *Gsdmd^cKO‐Nes^* cortices. *n* = 10 from 3 independent experiments). Error bars represent means ± SEMs; 2‐tailed unpaired *t*‐test; one‐way ANOVA with Dunnett's multiple‐comparison correction. ^*^ *p* < 0.05, ^**^ *p* < 0.01, ^***^ *p* < 0.001; n.s., not significant.

Given our previous data suggesting that the executioner of pyroptosis, GSDMD, plays an essential role in removing damaged cells from the developing cerebral cortex, we investigated how its absence specifically affects the process of shaping neurogenesis in the cerebral cortex. We electroporated GFP plasmids into the brains of E13 *Gsdmd^fl/fl^* and *Gsdmd^cKO‐Nes^* mice, and the cerebral cortex of *Gsdmd^cKO‐Nes^* mice exhibited an abnormal distribution of GFP compared to *Gsdmd^fl/fl^* mice, with a significant increase in GFP^+^ cells in the VZ/SVZ region and a significant decrease in GFP^+^ cells in the CP layer compared to *Gsdmd^fl/fl^* (**Figure** **2**A). There was a distinct increase in the number of GFP^+^ cells in the VZ/SVZ region, where neural progenitor cells are predominantly distributed (Figure 2B). We tested whether the significant increase in GFP^+^ cells was due to the promotion of neural progenitor cell proliferation. When the replicating population of uncommitted cells was examined at E15.5 (mid‐neurogenesis), there was a marked change in the angle of division. In dividing cells with a horizontal cleavage plane relative to the ventricular surface, we found a significant increase in dividing cells with a vertical cleavage plane (Figure 2C,D). Meanwhile, the preference of Sox2 in anaphase cells for horizontally aligned divisions was associated with an increase in stem cell self‐renewal.^[^ ^21^ ^]^ We immunostained NPCs for Sox2 and found that the number of Sox2‐positive cells was significantly increased in the *Gsdmd^cKO‐Nes^* VZ/SVZ zone (Figure 2E,G). Several studies identified radial glial cells (RGs) and intermediate progenitor cells (IPs) as important NPCs.^[^ ^22^ ^]^ Therefore, we investigated the expression levels of the intermediate precursor‐specific marker Tbr2 and the radiate glia‐specific marker Pax6, respectively. We found that Pax6 expression was significantly increased, and Tbr2 expression was slightly increased (Figure S5A–D, Supporting Information). This indicated that the absence of GSDMD mainly caused an increase in NPC proliferation. Next, we tested whether the deletion of GSDMD affected NPC differentiation by detecting the neuronal marker Tuj1 using immunofluorescence staining, finding that the deletion of GSDMD resulted in a dramatic decrease in neuronal differentiation (Figure 2F,H). We also quantified protein levels of Sox2 and Tuj1 markers, and the results were consistent (Figure 2I,J). We used pH3 to mark mitotically active cells and observed an increase in dividing cells upon loss of GSDMD (Figure S5E,F, Supporting Information). We labeled proliferating S‐phase progenitor cells by injecting BrdU 2 h beforehand and found that BrdU‐positive cells in the proliferative phase contained more γH2AX after GSDMD loss (Figure S5G,H, Supporting Information). Some studies have found that the accumulation of DNA damage can affect the cell cycle.^[^ ^13^ ^]^ To verify this, we injected pregnant mice with BrdU for 24 h and then analyzed the brains at E16, finding that the percentage of cells that exited the cell cycle (percentage of BrdU^+^Ki67^−^/BrdU^+^Ki67^+^ cells) was reduced in the absence of GSDMD (Figure 2K,L), indicating that an increase in cell proliferation was accompanied by a decrease in cell cycle exit. These findings provide evidence that GSDMD enhances mitosis in NPCs and inhibits neural differentiation.

The decrease in neuronal differentiation prompted us to examine what abnormalities occur in neurons and which cell types are abnormal. We first examined the morphology of neurons, we electroporated the GFP plasmid into the mice brain at E13 and isolated GFP‐positive primary neural progenitor cells. After in vitro differentiation for 4–6 days. We found that GSDMD deficiency led to abnormal dendritic morphology, and our observational analyses revealed a clear reduction in total dendritic length (Figure 2M,N). We performed long‐term IUE experiments from E13 to P0 in WT and cKO mice using the GFP plasmid to exclude the effect of the in vitro environment. The results showed that GFP cells almost always migrated to the upper layers of the cerebral cortex. However, GSDMD‐deficient neurons showed abnormal morphology and significantly reduced primary dendrites (Figure 2O,P). Immunofluorescence staining with layer‐specific markers at P0 showed a decrease in Satb2 and Tbr1‐positive neurons after GSDMD knockout, with no detectable change in Ctip2 neurons (Figure S5I,L, Supporting Information); this decrease was not due to apoptosis (Figure S5J,K, Supporting Information). These results provide evidence that GSDMD is required for neurogenesis and may be accompanied by specific fate changes that affect deep neurons.

### 2.4 Loss of GSDMD Causes Autistic‐like Behaviors in Pups and Adult Mice

**Figure 3:**
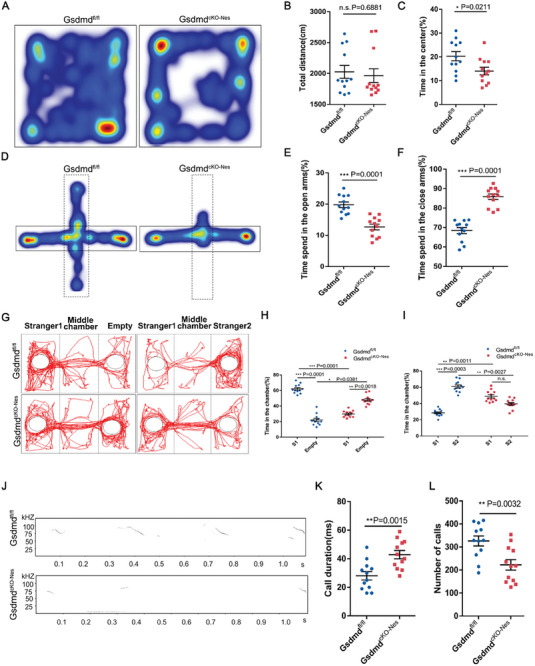
Lack of Gasdermin D activation drives autistic‐like behaviors in mice. A) Representative image of tracing pathway of *Gsdmd^fl/fl^* and *Gsdmd^cKO‐Nes^* mice in the open field test. B) The traveling distance was not different between *Gsdmd^fl/fl^* and *Gsdmd^cKO‐Nes^* mice within 5 min. *n* = 12 *Gsdmd^fl/fl^* and *Gsdmd^cKO‐Nes^* mice. C) Time in the center was reduced in *Gsdmd^fl/fl^* and *Gsdmd^cKO‐Nes^* mice within 5 min. *n* = 12 *Gsdmd^fl/fl^* and *Gsdmd^cKO‐Nes^* mice. D) Representative tracks in the Elevated‐plus maze test. E,F)Time spent in the open and closed arms. *n* = 12 *Gsdmd^fl/fl^* and *Gsdmd^cKO‐Nes^* mice. G) Representative tracks from “Stranger 1‐Empty” and “Stranger 1‐Stranger 2″ of *Gsdmd^fl/fl^* and *Gsdmd^cKO‐Nes^* mice. H) *Gsdmd^cKO‐Nes^* mice spent less time in the left chamber (Stranger 1) and more time in the right chamber (empty cage) when compared with *Gsdmd^fl/fl^*. *n* = 12 *Gsdmd^fl/fl^* and *Gsdmd^cKO‐Nes^* mice. I) *Gsdmd^cKO‐Nes^* mice spent less time for the novel partner (Stranger 2) and spent more time for Stranger 1 when compared with *Gsdmd^fl/fl^*. *n* = 12 *Gsdmd^fl/fl^* and *Gsdmd^cKO‐Nes^* mice. J) Representative USVs spectrogram in isolated pups. K,L) Significant difference of call duration and call numbers in USVs between isolated *Gsdmd^fl/fl^* and *Gsdmd^cKO‐Nes^* pups. n=12 *Gsdmd^fl/fl^* and *Gsdmd^cKO‐Nes^* pups. Error bars represent means ± SEMs; 2‐tailed unpaired *t*‐test; one‐way ANOVA with Dunnett's multiple‐comparison correction. ^*^ *p* < 0.05, ^**^ *p* < 0.01, ^***^ *p* < 0.001; n.s., not significant.

Activation of inflammasomes leads to the secretion of inflammatory factors as well as GSDMD‐mediated pyroptosis, both of which may affect neurodevelopment and behavior. We sought to determine whether GSDMD‐deficient mice have behavioral abnormalities and deficits. To assess the effects of GSDMD on healthy brain development, we first collected the brains of P0 mice and individual P8 mice and found that *Gsdmd^cKO‐Nes^* mice have relatively smaller brains and are significantly smaller in size compared with wide‐type mice (Figure S6A, Supporting Information). Next, we performed a series of behavioral tests on *Gsdmd^fl/fl^* and *Gsdmd^cKO‐Nes^* mice. In the open‐filed test, we found that *Gsdmd^cKO‐Nes^* mice spent less time in the central region than wide‐type mice, although they did not show a significant difference in total distance, suggesting reduced exploratory behavior in *Gsdmd^cKO‐Nes^* mice (**Figure** **3**A–C). To test whether mice developed deficits in exploratory ability, we used elevated cross‐maze and Y‐maze tests. We also observed that *Gsdmd^cKO‐Nes^* mice spent more time in the closed arm of the elevated cross‐maze than *Gsdmd^fl/fl^* mice (Figure 3D–F). The results of the Y‐maze test showed that *Gsdmd^cKO‐Nes^* mice were more inclined to enter the old arm without exploring the new arm compared to *Gsdmd^fl/fl^* mice (Figure S6B,C, Supporting Information), suggesting that GSDMD deficient mice exhibit anxiety‐like behavior and deficits in exploratory ability. To rule out the influence of locomotor activity, we performed a grip test with a rotation test to check motor abilities. The results showed no significant differences in grip strength or fall latency between *Gsdmd^fl/fl^* and *Gsdmd^cKO‐Nes^* groups, ruling out differences due to locomotion (Figure S6D,E, Supporting Information).

To investigate whether the mice exhibited abnormal social interactions, we conducted a three‐chamber social interaction in which wild‐type mice were more interested in stranger1 than in the empty cage, and *Gsdmd^cKO‐Nes^* mice were relatively less interested in stranger1. When stranger2 was placed in another empty cage, wide‐type mice tended to interact more with stranger2 mice than with the familiar stranger1 mice. However, *Gsdmd^cKO‐Nes^* mice showed no significant preference for interactions with stranger2 mice (Figure 3G–I). These results indicated that GSDMD‐deficient mice have impaired social interaction abilities. Based on these abnormal behaviors, we hypothesized that the deletion of GSDMD would cause mice to exhibit autistic‐like behaviors. We then recorded ultrasonic vocalizations (USV) from pups at postnatal day (P)6. When isolated from their mother, the *Gsdmd^cKO‐Nse^* pups produced a smaller number of calls and shorter call durations than the wide‐type pups (Figure 3J–L). Collectively, these behaviors suggest that GSDMD‐deficient mice exhibit autism‐like behaviors, such as anxiety, exploratory deficits, and social deficits.

### 2.5 Deletion of GSDMD Disruption of Electron Transport Chain Structure by Targeting Aifm3

**Figure 4:**
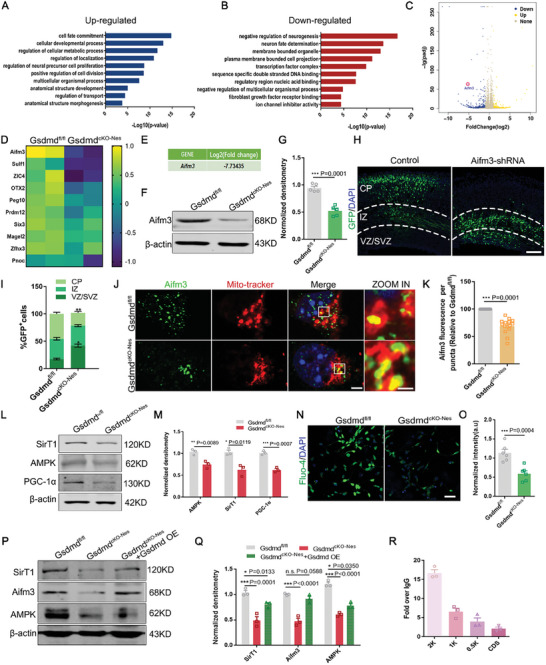
GSDMD regulates neurogenesis by targeting Aifm3 through the AMPK pathway. A, B) Gene Ontology (GO) analysis for downregulated or upregulated genes in the *GSDMD*‐depleted NPCs at E13 cerebral cortex. C)Differentially expressed genes (DEGs) were identified from the E13 forebrain of *Gsdmd^fl/fl^* and *Gsdmd^cKO‐Nes^* mice. Volcano plots illustrated the downregulated (blue) and upregulated (yellow) genes. D) Heat map was further analyzed of 10 differentially expressed genes from RNA‐Seq of the E13 brain. The Aifm3 gene was one of the significantly differentially expressed genes. E) RNA‐seq analysis showing that the Aifm3 mRNA expression level was dramatically reduced in the *Gsdmd^cKO‐Nes^* mice. F) Western blot analysis of Aifm3 protein levels in *Gsdmd^fl/fl^* and *Gsdmd^cKO‐Nes^* mice. G) The bar graph shows the normalized densitometry of Aifm3. *n* = 5 independent experiments. H) Electroporation of *Aifm3*‐shRNAs results in abnormal cell distribution in the developing neocortex. The electroporation was performed at E13, and the mouse brain was sacrificed at E16. I) Graph shows the percentage of GFP‐positive cells distributed in the VZ/SVZ, IZ, and CP. *n* = 5 independent experiments. J) Confocal immunofluorescence image of Aifm3 and Mito‐tracker in *Gsdmd^fl/fl^* and *Gsdmd^cKO‐Nes^* NPCs showing the different subcellular localization. Scale bar, 5 µm. The right zoom is a higher magnification image. Scale bars,1 µm. K) The bar graph shows the Aifm3 fluorescence intensity per puncta. The data are normalized to the *Gsdmd^fl/fl^*. *n* = 15 from 4 independent experiments. L) Western blot analysis of the expression levels of AMPK, SirT1, PGC‐1α in *Gsdmd^fl/fl^* and *Gsdmd^cKO‐Nes^* mice. M) The bar graph shows the normalized densitometry of AMPK, SirT1, and PGC‐1α. *n* = 3 independent experiments. N) Fluo‐4 AM staining of *Gsdmd^fl/fl^* and *Gsdmd^cKO‐Nes^* NPCs depicting intracellular calcium release. Scale bar, 20 µm. O) The graphs show the quantification of FLUO‐4 AM fluorescence in cultured cells. *n* = 5 independent experiments. P) Western blot analysis of NPCs from isolated *Gsdmd^fl/fl^* and *Gsdmd^cKO‐Nes^* mice were cultured in vitro for 2 days, after being treated with GSDMD‐overexpressing lentivirus for 24 hours. Q) The bar graph shows the normalized densitometry of SirT1, Aifm3, and AMPK. *n* = 3 independent experiments. R) N2a cells were transfected with Flag‐ PGC‐1α plasmid and cultured for 3 days before harvesting samples for Ch‐IP assay. ChIP‐qPCR showing PGC‐1α binds to the site of the Aifm3 promoter. *n* = 3 independent experiments. Error bars represent means ± SEMs; 2‐tailed unpaired *t*‐test; one‐way ANOVA with Dunnett's multiple‐comparison correction. ^*^ *p* < 0.05, ^**^ *p* < 0.01, ^***^ *p* < 0.001; n.s., not significant.

As we observed that GSDMD defects caused autistic behavior in mice, we next investigated the molecular mechanisms associated with the deletion phenotypes of GSDMD. To further explore the regulatory mechanisms of GSDMD in neurogenesis. Analysis of RNA sequencing results of cerebral cortical tissues from *Gsdmd^fl/fl^* and *Gsdmd^cKO‐Nes^* E13.5 mice showed that GO analyses of downregulated genes were enriched for cell fate commitment, cell developmental processes, and regulation of cellular metabolism processes. Simultaneously, upregulated genes were enriched in aspects associated with negative regulation of neurogenesis and regulation of plasma membrane‐bounded cell projections (**Figure** **4**A,B). Heatmaps and volcano maps show changes in the gene expression profiles of neural progenitor cells in the cerebral cortex of *Gsdmd^fl/fl^* and *Gsdmd^cKO‐Nes^* mice (Figure 4C; Figure S7A, Supporting Information). We further screened 10 genes whose expression was significantly downregulated upon GSDMD deletion (Figure 4D) and validated them by qPCR validation (Figure S7B, Supporting Information). We noticed the molecule apoptosis‐inducing factor mitochondria associated 3, Aifm3 (Figure 4E), localized mainly in the inner mitochondrial membrane as well as in the cytoplasm, was released from mitochondria as a caspase‐independent death effector and was involved in programmed cell death processes. We detected a significant decrease in protein expression levels (Figure 4F,G).

To verify whether GSDMD targets Aifm3 to regulate cortex neurogenesis, we further investigated the function of Aifm3 in cortical development and used IUE with *Aifm3*‐shRNA plasmids to examine the function of Aifm3, and similar to the results of the GFP^+^ cells in the VZ/SVZ regions that were significantly increased, GFP^+^ cells in the CP layer were clearly decreased (Figure 4H,I). Considering that Aifm3 is an important downstream target gene for GSDMD regulation, we tested whether Aifm3 overexpression rescued the abnormal distribution of GFP^+^ cells induced by GSDMD knockdown. Therefore, we co‐electroporated *GSDMD*‐shRNA with an *Aifm3* overexpression plasmid into the E13 mouse brain and found that plasmid overexpression rescued the aberrant distribution of GFP^+^ cells caused by GSDMD defects to some extent (Figure S7C,D, Supporting Information). Further study of the role of Aifm3 in the development of the cortex and immunofluorescence staining of the cerebral cortex after electroporation of the shRNA plasmid revealed that more PAX6^+^ GFP^+^ was observed in the VZ/SVZ region after *Aifm3* knockdown (Figure S7E,F, Supporting Information), demonstrating that *Aifm3* is an essential gene downstream of GSDMD in cortical neurogenesis.

We examined the localization relationship between Aifm3 and mitochondria, while mitochondrial expression of Aifm3 was significantly decreased after GSDMD knockout (Figure 4J,K). To further investigate the role of Aifm3 in neurogenesis under high replication stress, we infected NPCs with *Aifm3*‐shRNA lentivirus and stimulated them with bFGF treatment. Protein blotting showed that cleaved‐caspase‐1, was not affected by *Aifm3* knockdown, but there was a significant downregulation of cleaved‐caspase3; under bFGF stimulation, *Aifm3* knockdown affected neural progenitor cell apoptosis (Figure S7G,H, Supporting Information), which also explains the fact that apoptosis, another mode of regulatory cell death, was not increased when pyroptosis was inhibited in the previous work. These data suggest that both regulatory cell death pathways are present and necessary under high replication stress. Interestingly, the apoptosis‐inducing mechanism of Aifm3, which is based on cytochrome c release and caspase3 activation is consistent with our RNA sequencing analysis. Therefore, we suggest that the apoptosis‐inducing function of GSDMD is achieved by regulating *Aifm3* expression. Of note, literature has mentioned that GSDMD‐N and AMPK exist in a reciprocal regulatory relationship, DNA damage accumulation inhibition of AMPK signaling leads to a decrease in the expression of apoptosis‐inducing factors.^[^ ^23^ ^]^ Its downstream factor SirT1 responds to the stimulation of DNA damage through degradation and ubiquitination and it reduces apoptosis through the PGC‐1α mitochondrial pathway.^[^ ^24^, ^25^ ^]^ Therefore, we examined cortical NPCs after GSDMD deletion and found that the protein levels of AMPK, SirT1, and PGC‐1α were significantly reduced after GSDMD deletion (Figure 4L,M). To investigate how GSDMD knockdown inhibits the AMPK pathway, we performed a literature review and experiments and found that the pore formed by GSDMD mediates inward calcium flux, which upon encounter with a signaling stimulus triggers the endosomal sorting complex required for transport (ESCRT)‐dependent membrane repair and induces phosphatidylserine exposure.^[^ ^26^, ^27^ ^]^ Furthermore, the processing and release of *Aifm3* from the mitochondrial membrane requires the activation of CAMKII by calcium ions^[^ ^27^ ^]^ and the absence of GSDMD leads to perturbations in the intracellular environment with reduced calcium ion efflux and activation of calpain (Figure 4N,O), thus reducing the formation of complexes with its substrate CAMKII, which in turn reduces the formation of complexes with its substrate AMPK, and regulates intracellular and energy metabolism homeostasis, among others. To further confirm that GSDMD deletion affects mitochondrial protein synthesis through AMPK/SirT/PGC‐1α pathway, and to exclude the involvement of other mitochondrial‐related proteins, We analyzed the RNA sequencing data and found that AIFM3 was not the only mitochondrial protein that was downregulated, and other mitochondria‐related proteins had relatively insignificant changes (Figure S7I, Supporting Information). we constructed a PGC‐1α knockdown plasmid, packaged the lentivirus and then infected the NPCs, and extracted the RNA for RT‐PCR to detect, and found that only the expression of Aifm3 was significantly decreased by detecting the genes screened out from the sequencing data (Figure S7J, Supporting Information). Accordingly, we determined whether PGC‐1α binds to the promoter regions of Aifm3 using a chromatin immunoprecipitation assay. For the enrichment analysis, we designed 4 pairs of primers targeting different regions of Aifm3. Our results showed that PGC‐1α was significantly enriched in the Aifm3 promoter region located 2 kb from the transcriptional start site (Figure 4R). Taken together, our data suggest that GSDMD regulates the expression of the mitochondrial protein Aifm3 through the AMPK/SirT1/PGC‐1α pathway, thereby influencing the onset of apoptosis. we added rescue experiments by culturing WT and cKO neural stem cells in vitro and infecting them with GSDMD overexpression lentivirus, and found that transfection with GSDMD overexpression could rescue the expression of AIFM3 as well as AMPK to some extent by western blot (Figure 4P,Q).

### 2.6 AIFM3 Affects Mitochondrial Respiratory Chain Assembly

**Figure 5:**
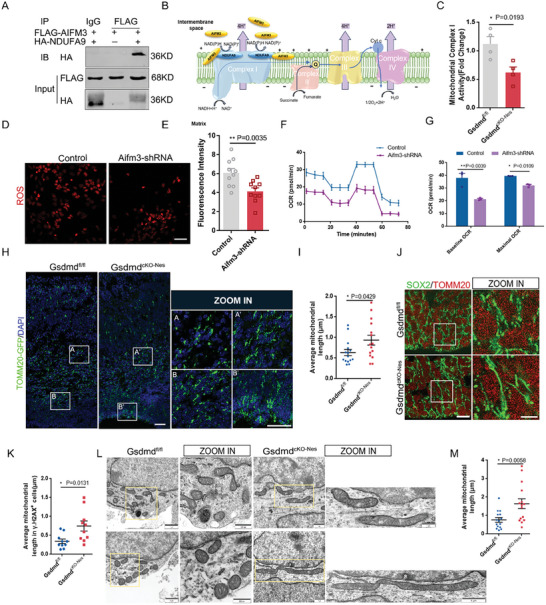
Aifm3 affects respiratory chain assembly. A) Co‐immunoprecipitation (CO‐IP) experiment was performed to detect the interaction between Aifm3 and Nudfa9 in N2a cells. N2a cells were transfected with FLAG‐ AIFM3 and HA‐NDUFA9 overexpression plasmids for 3d and harvested for CO‐IP assays. B) Schematic representation of the mitochondrial electron transport chain (ETC), Complex I, Complex II, Complex III, and Complex IV components of the Electron Transport Chain. The schematic shows Aifm3 interaction with Ndufa9 from Complex I. C) Mitochondria Complex I activity was measured from primary neural progenitor cells isolated from *Gsdmd^fl/fl^* mice and *Gsdmd^cKO‐Nes^* mice. *n* = 4 independent experiments. D) Confocal immunofluorescence image of ROS level labeled by DHE in control and Aifm3‐shRNA lentivirus‐infected NPCs. Scale bar,100 µm. E) Quantification showing the changed ROS level in control and Aifm3‐shRNA. *n* = 10 from 4 independent biological replicates. F) Measurement of oxygen consumption rate (OCR) of NPCs derived from E13 mice as control and then treated with Aifm3 shRNA lentivirus for 24 h in proliferation medium. G) Quantification of the Baseline and maximum OCR of NPCs. *n* = 4 independent experiments. H) The TOMM20‐GFP plasmid was electroporated into the E13 mouse brains of *Gsdmd^fl/fl^* mice and *Gsdmd^cKO‐Nes^* mice, and the mice were sacrificed at E16. Scale bars, 50 µm. The zoom in A, A′, B, and B′ are higher magnification images. Scale bars, 20 µm. I) Quantification of the mitochondrial length in *Gsdmd^fl/fl^* mice and *Gsdmd^cKO‐Nes^* mice. *n* = 30 from 4 independent experiments. J) Representative confocal images of mitochondrial morphology in SOX2^+^ cells in VZ of E16 coronal sections. Scale bars,20 µm. The right zoom is are higher magnification image. Scale bars,2 µm. K) The graph shows the length of mitochondria in SOX2^+^ cells in VZ of E13 coronal sections. *n* = 15 from 3 independent experiments. L) TEM image of the mitochondria at E16 from the *Gsdmd^fl/fl^* mice and *Gsdmd^cKO‐Nes^* mice. The scale is shown in the figure as shown. ZOOM IN panels show magnification of the area marked in the left panels. M) Quantification of the mitochondrial length in *Gsdmd^fl/fl^* mice and *Gsdmd^cKO‐Nes^* mice. *n* = 15 from 3 independent experiments. Error bars represent means ± SEMs; 2‐tailed unpaired *t*‐test; one‐way ANOVA with Dunnett's multiple‐comparison correction. ^*^ *p* < 0.05, ^**^ *p* < 0.01, ^***^ *p* < 0.001; n.s., not significant.

We previously found that Aifm3 localization on mitochondria was significantly reduced in cKO mice, and we next examined changes in expression throughout the cerebral cortex and found that expression was significantly reduced in cKO mice (Figure S7K,L, Supporting Information). To further investigate the abnormalities caused by changes in Aifm3 expression in mitochondria. We noticed several studies have indicated that AIF participates not only in the cell death pathway but also within the mitochondria, where it regulates complex I proteins in the mitochondrial respiratory chain to determine the rate of oxidative phosphorylation.^[^ ^28^ ^]^ Aifm3 is a structural homolog of AIF and shares mitochondrial localization. Therefore, we performed qPCR analysis of mitochondrial respiratory chain complexes I, II, III, IV, and V for the expression of key elements at the RNA level in *Gsdmd^fl/fl^* and *Gsdmd^cKO‐Nes^* brain cortices and found that it was the complex I component Ndufa9 that was significantly decreased (Figure S8A, Supporting Information). Based on this, we tested whether there was a direct interaction between Aifm3 and Ndufa9. We conducted immunoprecipitation experiments to verify the relationship between the two, which showed that FLAG‐tagged Aifm3 could pull down HA‐tagged Ndufa9 (**Figure** **5**A). It is suggested that Aifm3 affects the composition of mitochondrial respiration through interaction with the complex 1 component Ndufa9 (Figure 5B). Aifm3 is able to bind to the components of complex 1 and work together to complete the assembly of the respiratory chain, and whether the expression of Aifm3 affects the function of complex I. We then performed an assay of respiratory chain I activity and found that inhibiting the expression of Aifm3 affects complex I activity (Figure 5C). Given the established role of complex I as a major site for the production of ROS,^[^ ^29^ ^]^ Complex I‐mediated ROS levels were significantly reduced in infected NPC with *Aifm3‐*shRNA lentivirus (Figure 5D,E). GSDMD deletion resulted in reduced expression of Aifm3 affecting the assembly of respiratory chain complex I, which is recognized as the main site of ROS production.^[^ ^30^, ^31^ ^]^ Whereas OCR is mainly detected for mitochondrial respiratory chain complexes, the function of complex I was impaired by Aifm3. To address this point, we infected neural progenitor cells with Aifm3 knockdown plasmid for OCR detection and found the same significant reduction (Figure 5F,G). These data indicated that deletion of Aifm3 causes abnormal neurogenesis affects mitochondrial respiratory chain complex I assembly and reduces ROS production in neural progenitor cells. Based on these changes, we are able to explore new functions of GSDMD beyond cell death.

### 2.7 Mitochondrial Dynamics of GSDMD‐deficient NPCs were Impaired

Overall, our data propose that GSDMD can modulate Aifm3 to affect mitochondrial composition and redox processes. It has been reported in previous articles that mitochondria in neural stem cells maintain low ROS levels for extended periods during early development, which contributes to neural stem cell proliferation.^[^ ^32^ ^]^ This makes us curious about the role of mitochondria in development. The inflammatory activation of AIM2 has been shown to increase GSDMD binding to mitochondria and N‐terminal perforation of the mitochondrial membrane, inducing cell death.^[^ ^13^, ^33^ ^]^ Mitochondrial fission and fusion have been described to differentially affect neural stem cell self‐renewal.^[^ ^34^, ^35^ ^]^ We aimed to determine whether the aberrant neurogenesis induced by GSDMD deletion is associated with abnormal mitochondrial dynamics. We constructed a TOMM20‐GFP fusion protein that can label mitochondria in cells and electroporated it into E13 mouse brains and found that mitochondrial length was significantly increased in mice by GFP fluorescence analysis (Figure 5H,I). To verify this change, in vitro culture of NPCs isolated from the cortex of E13 mice, stained with Mito Tracker, and statistical analysis revealed a dramatic increase in mitochondrial length in *Gsdmd^cKO‐Nes^* mice (Figure S8B,C, Supporting Information). Transmission electron microscopy (TEM) of the cerebral cortex region of WT and cKO and showed that mitochondrial morphology changed, mitochondria exhibited an elongated morphology (Figure 5L,M).

Next, we tested the causes of the changes affecting mitochondrial dynamics. First, we examined the mitochondrial marker Tomm20 in *Gsdmd^fl/fl^* and *Gssdmd^cKO‐Nes^* mice and found no changes in protein levels (Figure S8F,G, Supporting Information), suggesting that the dynamics of mitochondrial division and fusion were altered. DRP1 is a major regulator of mitochondrial dynamics, and phosphorylation of DRP1 is the most widely studied post‐translational modification that regulates mitochondrial morphology, with phosphorylation occurring at 2 serine residues, ser‐616 and ser‐637.^[^ ^36^ ^]^ Apoptosis‐inducing factors are released following stimulation by DRP1 to initiate apoptotic signaling during mitochondrial changes.^[^ ^37^ ^]^ We examined the protein expression levels of DRP1 and DRP1ser‐637 and ser‐616 found that there was no difference in DRP1 and DRP1 ser‐616 protein levels, but there was a significant increase in ser‐637 phosphorylation (Figure S8F,G, Supporting Information), and phosphorylation of ser‐637 prevented DRP1 from translocating to the mitochondria to inhibit mitochondrial division.^[^ ^38^ ^]^ Next, we analyzed the subcellular distribution of DRP1 and performed immunofluorescence staining of DRP1 and Mito Tracker in NPCs in vitro. We found that DRP1 preferentially localized to the cytoplasm in *Gsdmd^cKO‐Nes^*, with significantly less accumulation in the mitochondria (Figure S8D,E, Supporting Information). These results suggest that the reduction of the mitochondrial membrane protein Aifm3 affects mitochondrial function and impairs the recruitment of DRP1 to mitochondria.

To test whether mitochondrial dynamics play a role in the determination of stem cell fate, we first examined Sox2^+^ cells in the VZ/SVZ region and found elongated mitochondria in Sox2‐positive cells compared with *Gsdmd^fl/fl^* (Figure 5J,K). While neural stem cells require more energy to proliferate, energy is metabolized differently in proliferating cells than in differentiated cells. In conclusion, GSDMD‐mediated reduction of Aifm3 not only affects mitochondrial electron transport chain structure but also leads to aberrant mitochondrial morphology.

### 2.8 Disruption of Pyroptosis Shifts Mitochondrial Metabolic Mode and Enhances Lactate Production

**Figure 6:**
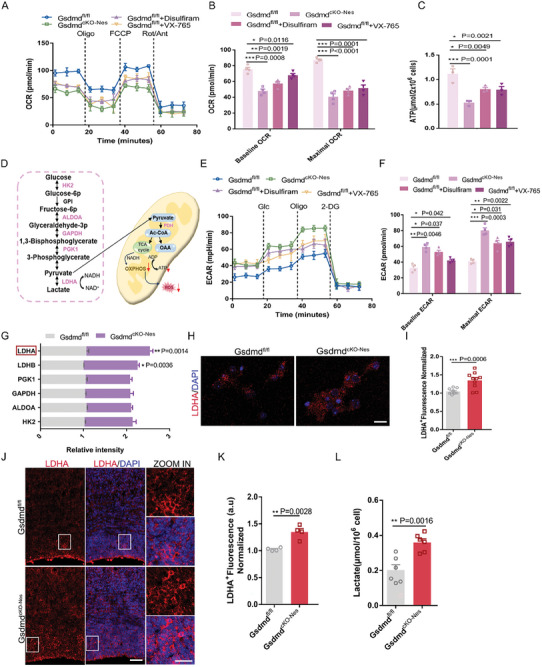
Disruption of pyroptosis shifts mitochondrial metabolic mode and enhances lactate production. A) Oxygen consumption rate (OCR) measurement of NPCs derived from *Gsdmd^fl/fl^* and *Gsdmd^cKO‐Nes^* (untreated or pretreated with VX‐756 (10 µM) or Disulfiram (0.3 µM)). B) Quantification of the Baseline and maximum OCR of NPCs. *n* = 4 independent experiments. C) ATP levels in NPCs derived from *Gsdmd^fl/fl^* and *Gsdmd^cKO‐Nes^* (untreated or pretreated with VX‐756 (10 µM) or Disulfiram (0.3 µM)). D) Schematic diagram of the glycolysis pathway (purple) and the mitochondrial oxphos including the TCA cycle. E) Extracellular acidification rate (ECAR) measurement of NPCs derived from *Gsdmd^fl/fl^* and *Gsdmd^cKO‐Nes^* (untreated or pretreated VX‐756 or Disulfiram). *n* = 4 replicates from 3 independent experiments. F) Quantification of the Baseline and Maximal ECAR of NPCs. G) qRT–PCR analysis of the representative glycolysis genes. The most significant between *Gsdmd^fl/fl^* mice and *Gsdmd^cKO‐Nes^* mice up‐regulated gene, LDHA, is indicated by the red box at the top. *n* = 4 independent experiments. H) Confocal images of NPCs derived from *Gsdmd^fl/fl^* mice and *Gsdmd^cKO‐Nes^* stained for LDHA and DAPI. Scale bar,10 µm. I) Quantification of the LDHA fluorescence intensity in *Gsdmd^fl/fl^* and *Gsdmd^cKO‐Ne^*. *n* = 10 replicates from 3 independent experiments. J) Brain sections of *Gsdmd^fl/fl^* mice and *Gsdmd^cKO‐Nes^* mice at E16 were immunostained with LDHA and DAPI. Scale bar,100 µm. The right zoom is higher magnification images. Scale bars,10 µm. K) The bar graph shows the LDHA fluorescence intensity in VZ/SVZ. The data are normalized to the *Gsdmd^fl/fl^*. *n* = 4 independent experiments. L) Lactate levels in NPCs isolated from *Gsdmd^fl/fl^* mice and *Gsdmd^cKO‐Nes^* mice. *n* = 6 independent experiments. Error bars represent means ± SEMs; 2‐tailed unpaired *t*‐test; one‐way ANOVA with Dunnett's multiple‐comparison correction. ^*^ *p* < 0.05, ^**^ *p* < 0.01, ^***^ *p* < 0.001; n.s., not significant.

During cell growth and proliferation, glycolysis and mitochondrial oxidative phosphorylation as the major metabolic pathways are co‐regulated by both internal and external signals, and glucose supplies fuel for both pathways. Mitochondrial defects resulting from decreased Aifm3 expression induced by GSDMD deletion during development are unclear regarding the metabolic characterization and function of mitochondria as energy factories in neural progenitor cells. Oxygen consumption (OCR) of NPCs in *Gsdmd^cKO‐Nes^* mice was then analyzed to assess mitochondrial respiration, *Gsdmd^cKO‐Nes^* NPCs consumed oxygen at significantly lower basal levels, resulting in reduced maximal respiratory capacity (**Figure** **6**A,B). In addition, we found that mitochondrial respiration capacity was similarly inhibited in cells treated with Caspase‐1 inhibitors and GSDMD pore formation inhibitors (Figure 6A,B). These data suggest that oxidative phosphorylation is inhibited in *Gsdmd^cKO‐Nes^*‐deficient neural progenitor cells and that this alteration is associated with cleavage of GSDMD and deletion of the downstream molecule Aifm3. Since Aifm3 inhibition affects the mitochondrial respiratory chain, we examined the ATP changes in different states. We found that decreased expression of Aifm3 caused defects in terms of a decrease in the level of ATP (Figure 6C). Inhibition of oxidative phosphorylation due to disruption of the TCA cycle suggests that glucose may be metabolized by glycolysis in proliferating neural progenitor cells (Figure 6D). We then examined the effect on glycolysis by detecting the glucose‐induced extracellular acidification rate (ECAR) and *Gsdmd^cKO‐Nes^* NPCs exhibited significantly higher ECAR under both baseline and stress conditions compared to WT (Figure 6E,F). We also found that the extracellular acidification rate was likewise significantly elevated in the inhibitor‐treated group, implying that the absence of GSDMD or abnormal oligomerization of GSDMD‐N leads to a defect in oxidative phosphorylation and a shift in cellular metabolism toward glycolysis. These data suggest that the absence of GSDMD‐mediated pyroptosis also has an impact on cellular metabolism. We further systematically examined the expression of glycolytic pathway genes in NSCs, including hexose kinase 2 (Hk2), aldolase A (Aldoa), glyceraldehyde‐3‐phosphate dehydrogenase (GAPDH), phosphoglycerate kinase1 (Pgk1), lactate dehydrogenase A (Ldha), and lactate dehydrogenase B (Ldhb) were predominantly expressed in NPCs at early developmental stages under symmetric proliferative division.^[^ ^39^ ^]^ We quantified cortical RNA in the brains of *Gsdmd^fl/fl^* and *Gsdmd^cKO‐Nes^* mice using qPCR and found that Ldha expression was higher in *Gsdmd^cKO‐Nes^* mice than in *Gsdmd^fl/fl^* mice (Figure 6G). Consistently, we found that Ldha expression in the VZ/SVZ region of *Gsdmd^cKO‐Nes^* mice was significantly higher than that of *Gsdmd^fl/f^* ^l^ mice using immunofluorescence staining (Figure 6J,K). The same difference in expression was observed in *Gsdmd^cKO‐Nes^* cells (Figure 6H,I). In addition, we examined the lactate level differences in *Gsdmd^fl/fl^* and *Gsdmd^cKO‐Nes^* cells medium and found that the lactate level of *Gsdmd^cKO‐Nes^* was notably higher than that of *Gsdmd^fl/fl^* mice (Figure 6L). Overall, our data confirm that GSDMD not only regulates mitochondrial morphology but also reprograms cellular energy metabolism patterns in cortex development.

### 2.9 Disruption of Pyroptosis Mediates Mitochondrial Dysregulation and GSDMD Regulates Neurogenesis Through the LDHA/RAC1/p38 MAPK Pathway

**Figure 7:**
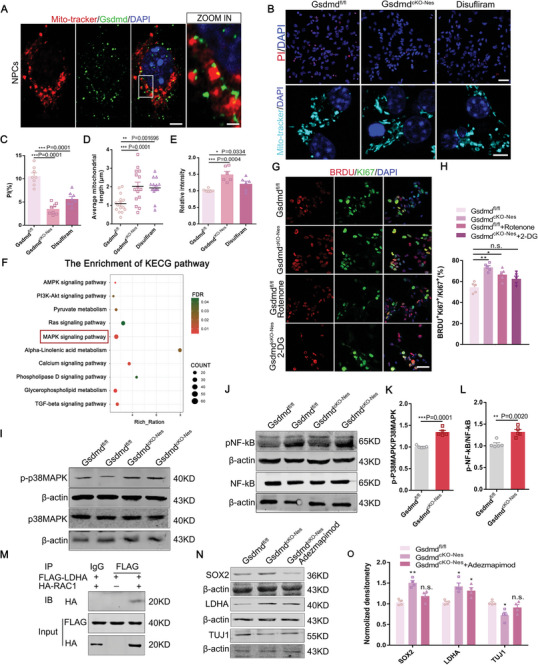
Disruption of pyroptosis mediates mitochondrial dysregulation and GSDMD regulates neurogenesis through the lactate pathway. A) Confocal immunofluorescence image of Mito Tracker and GSDMD in NPCs showing the subcellular localization of GSDMD in NPCs. Scale bar, 10 µm. The right zoom in higher magnification image. Scale bars,5 µm. B) Representative micrographs of PI, Mito Tracker, and DAPI staining of NPCs isolated from *Gsdmd^fl/fl^* and *Gsdmd^cko‐Nes^* mice and *Gsdmd^fl/fl^* treated with Disulfiram for 12 h. Up, scale bar,100 µm. Down, scale bar,20 µm. C) Quantification of the percentage of PI^+^ cells is shown in the histogram. *n* = 8 independent experiments. D) Quantitative analysis showed the length per mitochondria in *Gsdmd^fl/fl^* and *Gsdmd^cKO‐Nes^* and Disulfiram. *n* = 15 from 3 independent biological replicates. E) Quantification showing the changed IL‐1bβ level in *Gsdmd^fl/fl^*, *Gsdmd^cko‐Nes^* mice isolated NPCs. *n* = 6 independent experiments. F) KECG pathway analysis on the RNA‐seq dataset shows potential responses involving p38MAPK. G) Confocal immunofluorescence image of BrdU^+^Ki67^+^ in NPCs isolated from *Gsdmd^fl/fl^* and *Gsdmd^cKO‐Nes^* mice. Scale bar, 50 µm. H) Quantification showing 2‐DG inhibited the proliferation of NPCs. *n* = 5 independent experiments. I) Western blot analysis of the expression levels of p‐p38MAPK, p38MAPKin *Gsdmd^fl/fl^*, and *Gsdmd^cKO‐Nes^* mice. J) Western blot analysis of the expression levels of p‐p38MAPK, p38MAPK, pNF*‐κ*B, and NF*‐κ*B in *Gsdmd^fl/fl^* and *Gsdmd^cKO‐Nes^* mice. K,L) Statistics showing the increased ratio of p‐p38MAPK and p‐NF*‐κ*B in *Gsdmd^cKO‐Nes^* mice. M) Co‐immunoprecipitation (CO‐IP) experiment was performed to detect the interaction between LDHA and RAC1 in N2a cells. N2a cells were transfected with FLAG‐LDHA and HA‐RAC1 overexpression plasmids for 3d and harvested for CO‐IP assays. N) Western blot analysis of the expression levels of SOX2, LDHA, and TUJ1 in NPCs isolated from *Gsdmd^fl/fl^* mice and *Gsdmd^cKO‐Nes^* and *Gsdmd* ^cKO‐Nes^ treated with p38MAPK inhibitor Adezmapimod (0.5 µM) for 24 h. O) The bar graph shows the normalized densitometry of SOX2, LDHA, and TUJ1. *n* = 4 independent experiments. Error bars represent means ± SEMs; 2‐tailed unpaired *t*‐test; one‐way ANOVA with Dunnett's multiple‐comparison correction. ^*^ *p* < 0.05, ^**^ *p* < 0.01, ^***^ *p* < 0.001; n.s., not significant.

The deletion of GSDMD affects the structure of mitochondria and is a switch in the way of cellular metabolism. Thus, we are more curious about whether this change is due to the deletion of GSDMD or the reduction of GSDMD‐mediated pyroptosis. First, by immunofluorescence staining of mitochondria with Tomm20, we found the colocalization of GSDMD in neural progenitor cells (**Figure** **7**A). To test whether the absence of GSDMD contributes to this change in mitochondria, we added the GSDMD pore formation inhibitor disulfiram to the culture medium, finding that PI uptake was comparable between the disulfiram and *Gsdmd^cKO‐Nes^* groups, both of which showed a significant reduction, and that inhibition of pore formation slightly increased the length of mitochondria compared to *Gsdmd^fl/fl^* (Figure 7B,C,D). We also examined whether IL‐1β release in the cell culture medium was altered by pyroptosis (Figure 7E).

Different cell types will exhibit metabolic characteristics to satisfy the properties of different cells.^[^ ^40^ ^]^ There is increasing evidence that glycolysis is widespread in rapidly proliferating cells.^[^ ^41^ ^]^ We isolated and cultured primary neural progenitor cells in vitro with the addition of the electron chain transfer inhibitor rotenone, which causes mitochondrial complex I to uncouple from the electron transport chain and produce ATP via glycolysis, leading to lactate accumulation. The glycolysis inhibitor 2‐DG suppresses glycolysis by inhibiting the function of glucose‐6‐phosphate isomerase and hexokinase through the generation and intracellular accumulation of 2‐deoxy‐d‐glucose‐6‐phosphate (2‐DG6P).^[^ ^42^ ^]^ We found that inhibition of the respiratory electron chain promoted proliferation, whereas inhibition of glycolysis rescued the over‐proliferation of *Gsdmd^cKO‐Nes^* cells (Figure 7G,H). To further investigate the molecular mechanisms by which metabolic dysfunction regulates neurogenesis as a result of GSDMD deletion, we performed Kyoto Encylopedia or Genes and Genomes (KECG) pathway analysis on the RNA sequencing dataset, which showed that the genes were mainly enriched in the MAPK, Ras, and metabolic pathways (Figure 7F). We then investigated the signaling pathways regulating neurogenesis by GSDMD and found that the protein expression levels of p‐p38MAPK, p‐NF‐𝜅B were increased in *Gsdmd^cKO‐Nes^* mice (Figure 7I–L). Lactate was recently found to shuttle to provide energy requirements and regulate functional signaling during cortical development.^[^ ^43^, ^44^ ^]^ Previous studies have shown that Ldha interacts with GTPase‐Rac1, an activator of the p38MAPK pathway, in cancer cells.^[^ ^45^ ^]^ We examined the interaction between Ldha and Rac1 by immunoprecipitation and showed that FLAG‐tagged Ldha pulls down HA‐tagged Rac1 (Figure 7M), indicating that there is an interaction between the 2 and that Ldha regulates the p38MAPK pathway by interacting with Rac1. Apart from this, there was a significant upregulation of phosphorylated NF‐𝜅B (p65) in NPCs of *Gsdmd^cKO‐Nes^* mice thereby Ldha regulates the p38MAPK pathway to activate the transcription of NF‐𝜅B and orchestrates neurogenesis during embryonic development. We further tested whether increased p‐p38MAPK signaling may be responsible for the abnormal neurogenesis, and we cultured NPCs in vitro by adding adezmapimodan, an inhibitor of the p38MAPK pathway to the culture medium, finding that this inhibition rescued the impaired neurogenesis without affecting the expression of Ldha (Figure 7N,O). We confirmed the effects of pyroptosis deficiency in the developing cerebral cortex and distinguished the role of pyroptosis and its executor, GSDMD, in regulating mitochondrial structure, and further explored the altered cellular metabolic mode of NPCs in addition to GSDMD mediating pyroptosis, and we confirmed the activation of the neurogenesis signaling pathway via lactate signaling transmission (Figure S9, Supporting Information).

## 3 Discussion

Regulatory cell death plays a critical role in establishing proper development by causing substantial cell death during the peak developmental period.^[^ ^46^ ^]^ Our study provides compelling evidence that the peak of embryonic brain development coincides with the onset of pyroptosis, and demonstrates the ability of pyroptosis to respond to endogenous immune stress signals. Double‐stranded DNA breaks resulting from high‐intensity replication stress activate the AIM2 receptor, and the N‐terminal oligomerization of GSDMD punches holes in the cellular and mitochondrial membranes, causing cells to swell and rupture.^[^ ^33^ ^]^ We observed an accumulation of DNA damage in the VZ/SVZ area of the cortex in the absence of GSDMD. These findings suggest that GSDMD has a neuroprotective function in NPCs with heavy DNA damage, aiding in maintaining order in the cortical developmental environment. The data in this study provide evidence for novel cellular functions of GSDMD.^[^ ^47^ ^]^ Our observations indicated that in the absence of GSDMD, neural progenitor cell proliferation was promoted, whereas neuronal differentiation was inhibited. Our in vivo GFP tracing experiments further showed that the deletion of GSDMD resulted in reduced dendritic lengths and disorganized neuronal distribution across the cortical layers. This leads to developmental deficits in adult mice, such as anxiety‐like behaviors, social deficits, and other phenomena.

Cortical RNA sequencing at E13.5, revealed a significant decrease in Aifm3 expression in *Gsdmd^cko‐Nes^* mice compared to *Gsdmd^fl/fl^*. Interestingly, Aifm3 is mainly localized in the mitochondrial membrane and induces apoptosis. Zheng et al. reported that Aifm3 can be applied as a prognostic bio‐indicator for breast cancer patients.^[^ ^48^ ^]^ Our research has shown that Aifm3 knockdown results in impaired neurogenesis and oxidative state of the NPCs and indicated that Aifm3 interacts with Ndufa9, a component of complex I, in the electron transport chain of the inner mitochondrial membrane, disrupts the electron transport chain and inhibits the TCA cycle, which in turn inhibits oxidative phosphorylation. Besides this, we found that the absence of GSDMD affects the synergistic effect of pyroptosis and apoptosis by modulating PGC‐1α to alter the expression of the mitochondrial membrane protein Aifm3.

Mitochondria are indispensable for the proliferation and differentiation of stem cells and fate determination,^[^ ^49^ ^]^ which play important roles in cell death, energy metabolism, and signaling pathways,^[^ ^50^ ^]^ we found that the length of mitochondria in the cortex of GSDMD‐deficient mice was significantly increased. We searched the literature for this phenomenon and found that high molecular weight GTPases are central elements of mitochondrial dynamics. Among them, DRP1 is a key molecule in the mitochondrial division, whose defective function affects embryonic neuronal development.^[^ ^51^ ^]^ Our data indicate that this alteration was due to an increase in DRP1‐ser637, resulting in reduced translocation of DRP1 from the cytosol to the plasma membrane, inhibiting activated phosphorylation is thought to result from an intracellular ion imbalance following GSDMD deletion.

Many reports and databases have reported that the levels of metabolites detected in neurons of the brain are altered in patients diagnosed with autism spectrum disorder (ASD). About 5% of children with ASDs have mitochondrial dysfunction. Mitochondrial dysfunction impairs energy‐dependent physiological processes such as neurodevelopment and neuroplasticity, leading to autism.^[^ ^52^, ^53^ ^]^ Approximately one‐third of children with autism have been reported to have metabolic abnormalities and elevated levels of many mitochondrial biomarkers.^[^ ^54^ ^]^ A subgroup of individuals with ASDs have underlying mitochondrial dysfunction and altered fatty acid metabolism, as evidenced by higher levels of lactate, and pyruvate, and the prevalence of mitochondrial disease.^[^ ^55^ ^]^ GSDMD leads to severe morphological damage, disruption of electron transport, induction of reactive oxygen species, and mitochondrial autophagy, among others. In the central nervous system, dysfunctional mitochondria can induce inflammatory responses causing neuronal abnormalities. Leading to reduced efficiency or loss of ETC production in the mitochondrial electron transport chain, disturbed cellular energy metabolism, and triggering a variety of neurological disorders such as autism, depression, or Parkinson's disease. And our study confirms that GSDMD deficiency leads to metabolic disorders during neural stem cell development by affecting the way mitochondria metabolize energy. And is associated with autism‐like behavior in adult mice after birth. Mitochondrial defects resulting from decreased Aifm3 expression induced by GSDMD deletion during development are unclear regarding the metabolic characterization and function of mitochondria as energy factories in neural progenitor cells. We used Metabolic Flux assays to analyze the metabolic states of neural progenitor cells. We also identified the differential expression of Ldha, the enzyme encoding the transformation of pyruvate to lactate. Increasing evidence indicates that lactate is not only a metabolite but also a versatile signaling molecule.^[^ ^43^, ^56^ ^]^ A recent study indicated that Ldha is essential for neurogenesis to regenerate NADH^+^ via NADH to sustain anaerobic glycolysis, and high levels of lactate promote mitochondrial elongation;^[^ ^39^ ^]^ the increase in lactate levels and its secretion herald its potential as a signaling molecule to regulate neurogenesis. Coincidentally, we have found that up to ≈30% of children with autism have elevated plasma lactate.^[^ ^57^ ^]^ The combination of data from children with autism and our findings on metabolism and mitochondrial dysfunction may lead us to the assumption that the identification of biomarkers of these impairments in brain development will facilitate the development of personalized therapies for children with autism.

Our results suggest that pyroptosis does slightly influence changes in mitochondrial ROS levels, as observed using a pore‐forming inhibitor of pyroptosis disulfiram. Therefore, we raised questions about the imbalance in mitochondrial dynamics and aberrant neurogenesis after GSDMD deletion, and whether other signaling pathways are involved in regulating neurogenesis during development. Combined with sequencing analysis, we identified the signaling mechanism by which lactate acts as a signaling molecule to regulate neurogenesis. Recent studies have shown that Ldha interacts with Rac1, a GFP enzyme of the Rho family, to regulate cancer cell proliferation.^[^ ^45a,b^ ^]^ Our data also show that Ldha interacts with Rac1 in neural progenitor cells. Rac1 acts as an upstream activator of p38MAPK,^[^ ^58^ ^]^ regulates neurogenesis of the p38MAPK pathway, and promotes the phosphorylation of NF‐𝜅B.

In summary, our study indicated that pyroptosis during cortical development occurs in response to high levels of replicative stress and contributes to the elimination of damaged neural progenitor cells. Disruption of the pyroptosis pathway causes imbalances in DNA accumulation and mitochondrial dynamics, resulting in autism‐like behaviors. Our study expands the understanding of the molecular functions of GSDMD beyond pyroptosis. The absence of GSDMD is involved in the homeostasis of mitochondrial dynamics and the crosstalk between pyroptosis and apoptosis, which reprogrammed the metabolic profile of neural stem cells during embryonic development. Our results demonstrate that GSDMD is not only a cleaner during cortical development but also a guardian that maintains the balance of cellular metabolism and preserves the normal establishment of the nervous system. The findings may lead us to predict that biomarkers of these impairments in brain development will facilitate the development of personalized therapies for neurological disorders caused by innate immune injury and neurodevelopmental disorders such as autism.

## 4 Experimental Section

### Mice

Gsdmd floxed mice (B6.B6CB‐Gsdmdtm1.2Tshir/Ms) were obtained from the RIKEN BRC. *Nestin*‐Cre mice (B6. Cg‐Tg(Nes‐cre)1Kln/J) were provided by the Jackson Laboratory. To generate *Gsdmd^cKO‐Nes^* mice, *Gsdmd^fl/fl^* mice were crossed with the *Nestin*‐Cre mice line. C57BL/6 mice (8−10 weeks of age) and pregnant mice used for in‐utero electroporation were purchased from Vital River Laboratories (Beijing, China). All mouse feeding and experiments were approved and conducted by the relevant guidelines and regulations of the Experiment Animal Center of the Institute of Zoology, Chinese Academy of Sciences (IOZ20180037).

### Comet Assay

The Comet assay procedure was performed as described previously.^[^ ^13^ ^]^ Briefly, using the Oxiselect Comet Assay Kit (Cell‐Biolabs Inc.). Cells are gently pipetted into single cells, 1x10‐6 cells are mixed with prepared low‐melting agarose and dropped on agarose‐coated slides, treated for gel electrophoresis, dried and stained with fluorescent dyes, and subsequently observed with fluorescence microscopy. Confocal images were captured by Carl ZeissLSM880 and analyzed by CASP Lab software.

### BrdU Labeling

For NPC proliferation analysis, E16 pregnant mice were injected with BrdU (50 mg k^−1^g) 2 h before brain harvesting. For analysis of cell cycle exit, E15 pregnant mice were injected with BrdU (50 mg k^−1^g) 24 h before brain harvesting.

### Plasmid Constructs

Mouse *Aifm3* and *LDHA* were acquired by PCR and cloned into the pCDH‐copGFP vector with 3Flag‐tag. Mouse *Ndufa9* and *Rac1* were amplified and cloned into the pCDH‐copGFP vector with 3HA‐tag. Mouse TOMM20‐GFP was acquired by PCR and cloned into the pCAG‐copGFP vector. The PCR primer for cDNA is as follows:

*Aifm3*‐Forward 5′‐ CAA CCG CAA AGT GAACAT TCC −3′;
*Aifm3*‐Reverse 5′‐ TCC AGA GGT AGG GCA CAG −3′;
*LDHA*‐Forward 5′‐ GCAACCCTCAAGGACCAGCTGAT −3′;
LDHA‐Reverse 5′‐ TTAGAACTGCAGCTCCTTCTGGA −3′;
RAC1‐Forward 5′‐ CAGGCCATCAAGTGTGTGGTGGT −3′;
RAC1‐Reverse 5′‐ TTACAACAGCAGGCATTTTCTCT −3′;
NDUFA9‐Forward 5′‐ GCGGCCGCCGTCCGCTTTCGGGT −3′;
NDUFA9‐Reverse 5′‐ CTAATAGTTGACTGTCTTGGCAG −3′;
TOMM20‐GFP‐Forward 5′‐ GCCCGGGATCCACCGGTGCCACC‐3′;
TOMM20‐GFP‐Reverse 5′CCATGCTGGCGACCGGTTCCAC
ATCTTCAGCCAA −3′;

The sequences of shRNAs for *GSDMD* and *AIFM3* were cloned into pSicoR‐GFP vector. The sequences were as follows:

GSDMD ‐shRNA: GGTGAACATCGGAAAGATTTT
AIFM3 ‐shRNA: CATAAGTTCCAGGTGAAGATT
AIM2 ‐shRNA: CACGTTCTTTGAGGTGTCAAA
PGC‐1α‐shRNA: TCCCAGGATTAGTAAACTGAA
Human GSDMD sgRNA was cloned into the V2 vector.

The sequences were as follows:

hGSDMD‐sgRNA: TAATGAAAACTAGCCCCCGT

### Cell Cultures

Human embryonic kidney 293T cells (HEK293FT) mouse neuroblastoma N2a cells (ATCC CCL‐131) were cultured in high glucose DMEM (Gibco) and supplemented with 10% fetal bovine serum (FBS) (Gibco) and 1% penicillin/streptomycin (Invitrogen). H9 human ES cells were cultured in Essential 8 medium (Thermo). The cells were maintained on Matrigel‐coated six‐well plates (Corning), changed the medium once a day.

### Live Imaging Experiments

For live imaging experiments in *vivo*, the procedure was performed as described previously.^[^ ^59^ ^]^ Pre‐cool the medium DMEM/F12 in advance, Harvested the brains of E13 mice and glued them to a flat specimen base provided in the vibratome, prepared and sectioned 300 µm samples in the vibratome chamber filled with DMEM/F12 added PBS at room temperature. And adjusted lower speed and higher frequency. The freshly cut live slices are quickly transferred to a 15‐mm glass‐bottom culture dish (Nest) with proliferation medium. Quickly transferred the culture dish into the live cell culture confocal microscope. Live imaging experiments were performed using Andor Dragfly 200 scanning confocal microscope equipped and analyzed with ImarisViewerX64 9.5.1.

For live imaging experiments in vitro, to detect the morphology of pyroptotic cells, we treated cells in a 15‐mm glass‐bottom culture dish (Nest) as described. Pyroptotic cells in the static brightfield were captured using an Andor Dragfly 200 microscope and analyzed with ImarisViewerX64 9.5.1.

PI prolonged uptake experiments were captured and analyzed using an IncuCyte S3 2018C.

For the time‐lapse experiments, each field was imaged every 4–8 min at 20× or 40× magnification. All displayed images were selected from at least 3 random fields of view.

### Primary Neural Progenitor Cell Culture

Neural progenitor cells were isolated from the cerebral cortex of E13.5 mouse embryos and digested in papain for 5 min at 37 °C. Papain was then removed and washed 3 times with DMEM. After removing the supernatant, the cells are suspended in a fresh medium and filtered with a 70 mm filter membrane, centrifuged at 1000 rpm for 5 min, and resuspended. For the proliferation, primary NPCs were seeded 6‐well plate or 24‐well culture plate, which was coated with Poly‐d‐Lysine(10 µg mL^−1^) (Sigma) and Lamin (5 µg mL^−1^) (Invitrogen). The NPCs proliferation medium was composed of 50% DMEM/F12 medium (Invitrogen) and 50%Neurobasal medium (Invitrogen), supplied with 0.5% GlutaMAX (Invitrogen), 2% B27 supplement (without VA) (Invitrogen), 1% nonessential amino acids (Invitrogen), 5 ng mL^−1^ basic fibroblast growth factor (bFGF, 10 ng mL^−1^, Invitrogen), 2.5 ng mL^−1^ epidermal growth factor (EGF, 10 ng mL^−1^, Invitrogen), 1% penicillin/streptomycin (Invitrogen). The NPCs differentiation medium was composed of low glucose Dulbecco's Modified Eagle Medium (DMEM) (Gibco) and supplied with 10% fetal bovine serum (FBS) (Gibco), 2% B27 supplement (with VA) (Invitrogen) and 1% penicillin/streptomycin (Invitrogen).

### Generating Human Neural Precursor Cells

The procedure to generate human neural precursor cells(NPCs) from human embryonic stem cells is described previously.^[^ ^60^ ^]^ Briefly, after the density of H9 cells reaches 70%−80% in the culture plate, the culture medium was changed to NPCs differentiation medium consisting of 50% DMEMF/12 medium (Gibco), 50% Neurobasal medium (Gibco), 0.5% Gluta MAX (Invitrogen), 2% B27 supplement whit out VA (Invitrogen), 1% N2 supplement (Thermo Fisher), 10 ng mL^−1^ human LIF (Millipore), 4 × 10^−6^ m CHIR99021 (Stemgent), 3 × 10^−6^ m SB431542 (Tocris), 2 × 10^−6^ m dorsomorphin (Selleck), 0.1 × 10^−6^ m compound E (EMD Chemicals). After 5 days, single cells were dissociated with Accutase (Thermo Fisher) and seeded onto Metrigel‐coated plates. The cells were then cultured in human NPCs proliferation medium containing 50% Neurobasal medium (Gibco), 50% DMEMF/12 medium (Gibco), 0.5% GlutaMAX (Invitrogen), 2% B27 supplement without VA (Invitrogen), 1% N2 supplement (Invitrogen), 10 ng mL^−1^ hLIF (Millipore), 3 × 10^−6^ m CHIR99021, and 2 × 10^−6^ m SB431542. The medium would be changed every 2 days.

### Lentivirus Production and Infection

GenEscort II(Wisegen) was used to transfect lentiviral plasmids (Addgene) and lentiviral DNA plasmids (pSicoR, PCDH) into HEK293T cells. The culture medium was changed 8 h after transfection, and then at 24, 48, and 72 h, the culture medium supernatants were collected with lentivirus. For lentiviral infection, the virus was mixed with the medium in the presence of 4 mg mL^−1^ Polybrene and centrifuged at 3000 rpm for 5 min and added to the 6‐well plate or 24‐well plate to infect cells, and culture medium was exchanged 8 h later.

### Chromatin Immunoprecipitation

The transfected cells were maintained in 1% (1 × 10^−1^ m NaCl, 0.5 × 10^−3^ m EGTA, pH 7.5, 10 × 10^−4^ mm EDTA, 5 × 10^−2^ m HEPES‐KOH) fresh paraformaldehyde solution, crosslink by 10 min at room temperature. The reaction was then stopped by adding 2.5 m glycine and the cells were washed with cooled PBS 3 times. The cells were harvested in Lysis Buffer 1 (5 × 10^−2^ m HEPES‐KOH (pH 7.5), 14 × 10^−2^ m NaCl, 0.5% NP‐40, 10% glycerol, 1 × 10^−3^ m EDTA, 1× PMSF, 0.25% and Triton X‐100) for 15 min, and the lysate was separated at 5000 rpm, 4 °C for 10 min and pelleted in Lysis Buffer 2 (2 × 10^−1^ m NaCl, 1 × PMSF, 5 × 10^−4^ m EGTA, 1 × 10^−3^ m EDTA,1 × 10^−2^ m Tris‐HCl (pH 8.0)). At room temperature, the cells were vortexed gently for 10 min. Samples were sonicated with sonicated (Scientz‐IID) in Lysis Buffer 3 (5 × 10^−2^ m Tris‐HCl (pH 8), 1× PMSF, 1 × 10^−2^ m EDTA, 1% SDS). Samples were then incubated overnight at 4 °C with 50ul Protein A beads or anti‐HA beads. Beads were washed with low salt solution 3 times the next day (1% Triton X‐100, 20 × 10^−4^ m EDTA, 0.1% SDS, 1.5 × 10^−1^ m NaCl, Tris‐HCl (pH 8)), 3 times with high salt solution (1% Triton X‐100, 20 × 10^−2^ m EDTA, 5 × 10^−1^ m NaCl, 0.1% SDS, Tris‐HCl (pH 8)), and incubated at 65 °C overnight. TIANamp Genomic DNA Kit was used to extract the genome. and the DNA was quantified by RT‐PCR. The sequences of the primers used for the RT‐PCR are listed in Supplementary Table S2 (Supporting Information).

### Western Blotting and Co‐Immunoprecipitation

For western blotting, brain tissues/cells were lysed in RIPA lysis buffer (Solarbio) containing 1% PMSF, and 1% protease inhibitor cocktail. After centrifugation at 4 °C for 15 min, 12 000 rpm, the supernatant was collected and the concentration was determined by BCA. The sample was separated by 12% or 15% SDS‐PAGE gel and was transferred to the nitrocellulose (NC) membrane. The membrane was incubated with primary antibody overnight at 4 °C after blocking with 5% milk or 5% BSA for 1 h at room temperature. On the secondary day, after washing with PBST (0.05% Tween‐20 in 1 m PBS) 3 times, the secondary antibody is incubated for 1 h at room temperature. Visualization and analysis of the bands are performed by the Image Studio Ver 5.2 software.

For co‐immunoprecipitation, protein samples were lysed in co‐IP lysis buffer (Beyotime Biotechnology). The buffer contains 1% PMSF and 1% protease inhibitor cocktail. After centrifugation, the supernatant was incubated with 25 mL anti‐Flag or HA‐tag magnetic beads (MBL) at 4° overnight. The following day, they were washed 3 times with pre‐cold 0.02% PBST (Tween 20) and boiled for 5 min with loading buffer. Western blot was used to analyze the protein.

### Immunocytochemistry

Mouse brains were removed and fixed with 4% paraformaldehyde for 24 h, and dehydrated in 30% sucrose for 24 h. Brains were then embedded in Tissue‐Tek OCT and sectioned into 15 µm brain cryosections using a freezing microtome (Leica, CM1950, Germany). Brain sections or cells cultured in a 24‐well culture plate were fixated with 4% paraformaldehyde for 30 min, followed by three 10 min washes with PBS that included 1% Triton X‐100 (1%PBST). Then block with 5% BSA (in 1% PBST) for 1 h at room temperature. Cultured cells were washed 3 times for 10 min each with PBS that included 0.1% Triton X‐100 (0.1% PBST). Followed by blocking with 5% BSA (in 0.1% PBST) for 1 h at room temperature. All samples were incubated with the primary antibodies at 4 °C for one night. The next day, brain slices or culture cells were incubated with the secondary antibody for 1 h, then washed 3 times for 10 min with 1% PBST or 0.1% PBST. After this, the samples were incubated 40,6‐diamidino‐2‐phenylindole (DAPI 2 mg mL^−1^; Sigma) in PBS for 5 min at room temperature. Caspase‐1 activation was measured using the FAM‐FLICA Caspase‐1 Assay Kit (ImmunoChemistry Technologies). The assay was performed following the manufacturer's instructions. Related images are obtained using IncuCyte S3 2018C. Confocal images were obtained using Carl ZeissLSM880 or Leica Stellaris confocal microscope and analyzed using ZEN, LAS X Office, and ImageJ software.

### Evaluation of Mitochondrial Membrane Potential

Mitochondrial membrane potential (MMP) was analyzed by staining with JC‐1(Beyotime). Based on the manufacturer's instructions, added JC‐1 staining working solution and incubated for 20 min at 37 °C incubator. Then discard the supernatants and wash them 2 times with JC‐1 staining buffer (1X). Replaced with 2 mL cell medium and captured picture under confocal microscope. ImageJ software was used to analyze the images.

### DHE Detection of ROS Levels

Added ROS Fluorescent Probe‐DHE (10 µM mL^−1^) (Vigorous) into the neural stem cells proliferation medium, incubated at 37 °C for 30 min, Images were acquired with Carl ZeissLSM880 and analyzed using ZEN and ImageJ software.

### Pyroptosis Assay

Cell pyroptosis measurements were as previously described.^[^ ^61^ ^]^ Briefly, cells were seeded in 24‐well pre‐coated plated and cultured overnight. The cells were stained with a fluorescence staining solution containing Hoechst 33 342 (5 µg mL^−1^) and PI (2 µg mL^−1^). Images were acquired with Carl ZeissLSM880 and analyzed using ZEN and ImageJ software.

### In Utero Electroporation

The detailed IUE protocols have already been performed.^[^ ^62^ ^]^ The pregnant female mice were anesthetized by pentobarbital sodium (70 µg g^−1^) and the fetus was taken out, the control and target plasmids DNA mixed with 0.2% Fast Green (Sigma) were injected into the lateral ventricle of the fetal mice using a glass micropipette. The fetal brain was electroporated using an electroporator (BTX ECM830) with 5 mm diameter electrodes with five 50 ms pulses at 40 V with a 950 ms interval. After electroporation, sacrificed brain at different points in time.

### Lactate Assay

The level of lactate was measured using an L‐LA assay kit (Solarbio) according to the instructions of the manufacturer. In brief, the primary neural progenitor cells were seeded into 6‐well pre‐coated plates (Costar) at a population density of 5 × 10^6^ cells and cultured overnight. On the second day after the extraction solution was added, the cells were broken by the ice bath ultrasonic wave and centrifuged, and the supernatant was collected for assaying lactate levels.

### Lactate Dehydrogenase (LDH) Assay

Cell death is measured with different treatments by quantifying LDH released by cells in the supernatant using the CytoTox 96 Non‐Radioactive Cytotoxicity Assay kit (Promega) following the manufacturer's instructions.

### Metabolic Assays

The oxygen consumption rate (OCR) and extracellular acidification rate (ECAR) of primary neural progenitor cells were measured using a Seahorse XFe 96 Extracellular Flux Analyzer (Seahorse Bioscience) in accordance with the manufacturer's protocol. In brief, the cells were seeded into XF96‐well plates (1 × 10^4^ cells per well) (Agilent Technologies) and cultured overnight. The cells to be treated were added to VX765 (25 µM) or disulfiram (0.5 µM) 2 h before the assay. Cultured cells were washed twice and incubated in XF Assay Medium before assaying. OCR was measured by sequentially injecting 1 µM oligomycin, 0.25 µM FCCP, and 0.5 µM rotenone/antimycin A into the wells of the culture microplate at the time points indicated for OCR analysis using the Mito Stress Test Kit (Seahorse Bioscience). ECAR was measured using the XF Glycolytic Stress Test Kit (Seahorse Bioscience) by sequential injection of 10 mM glucose, 1 µM oligomycin and 100 mM 2‐DG into the wells of the culture microplate at the time points indicated for ECAR analysis. Seahorse XF 96 Wave software was used to analyze the data.

### Mitochondrial Complex I Activity Assay

The ratio of mitochondrial complex I activity was measured using a Mitochondrial Complex I Activity Assay kit (Bioss) according to the instructions of the manufacturer.

### Electron Microscopy Analysis

Electron microscopy procedures were performed as described previously.^[^ ^63^ ^]^ Briefly, mice were anesthetized and perfused with cacodylate buffer followed by 2% glutaraldehyde/2% paraformaldehyde. Mice brain blocks were cut into several 1 mm^3^ sample pieces using a double‐sided blade. The tissue blocks were postfixed for 12–24 h at 4 °C in the fixative mixture. Tissues were then first immersed in 1% OsO4 and 1.5% potassium ferricyanide aqueous solution at 4 °C for 2 h. Samples were infiltrated in graded mixture (8:1, 5:1, 3:1, 1:1, 1:3,1:5,1:8) of acetone and SPI‐PON812 resin (19.6 mL SPI‐PON812, 6.6 mL DDSA and 13.8 mL NMA), then changed pure resin. Finally, tissues were embedded in pure resin with 1.5% BDMA and polymerized for 12 h at 45 °C, 48 h at 60 °C. The ultrathin sections (70 nm thick) were sectioned with a microtome (Leica EM UC6), double‐stained by uranyl acetate and lead citrate, and examined by a transmission electron microscope (FEI Tecnai Spirit120kV) with the EMSIS CCD camera (VELETA). After obtaining, the ultrathin sections were stained by uranyl acetate and lead citrate, and examined by a transmission electron microscope.

### Quantitative Real‐Time PCR Analysis

Total RNA from mouse cerebral cortex or cells was extracted with TRIzol (Invitrogen) following the manufacturer's directions, and cDNA was obtained by the FastQuant RT Kit (TIANGEN). Real‐time PCR assays were performed using the SuperReal PreMix Plus (SYBR Green I) Kit (TIANGEN) on the ABI 7500 real‐time PCR system (Applied Biosystems). The sequences of the primers used for the real‐time PCR are listed in Supplementary Table S1 (Supporting Information).

### Animal Behavioral Experiments

All mouse feeding and experiments were approved and conducted by the relevant guidelines and regulations of the Experiment Animal Center of the Institute of Zoology, Chinese Academy of Sciences. The rearing environment was a 12‐h day/night cycle, SPF grade, and the mice for behavioral studies were separated from the experimental mice in cages of 3 mice each after genotype identification. Mice aged 8–12 weeks were selected for behavioral experiments based on birth time.

### Ultrasonic Vocalizations (USVs)

The USVs test was conducted as described above,^[^ ^64^ ^]^ the pups of *Gsdmd^fl/fl^* or *Gsdmd^cKO‐Nes^* were separated from their mother at the P6 stage, and USV emissions were recorded using Avisoft SASLab Pro (Version 5.2, Germany) and writing the number on the pup's abdomen and placing it in the isolation container for 5 min. The microphone was placed on top of the anechoic chamber and the pups were placed into the isolation container of the anechoic chamber while recording, and the isolation container was wiped with alcohol and air‐dried after each recording. The recording frequency is 15–180 kHz with a flat frequency response (±6 dB) between 25 and 140 kHz. The fast Fourier transform was applied as (512 FFT length, Hamming window, 100% frame, and 75% overlap of the time window). The spectrograms were 488 Hz and 0.512 ms in frequency and time resolution, respectively. Call detection was recorded with a hold time of 10 ms, a high‐pass filter of 30 kHz, an amplitude threshold of 40 dB, and a noise reduction filter of 40 dB. Our experiments were conducted from 9:00–16:00. Avisoft SASLab Pro software (version 5.2, Germany) was used to calculate call duration and call numbers.

### Open Field Test

Mice (8–12 weeks old) were individually placed in square boxes (40 × 40 × 40), and the behavioral activities of mice in the observation area were recorded for 5 min using the Etho vision XT14 behavioral analysis software, and statistical analysis was performed for the total distance and time spent in the central area of the box.

### Elevated‐Plus Maze

The Elevated‐Plus Maze consists of 2 opposing open arms (40 × 9.5 cm) and closed arms (40 × 9.5 cm) that intersect to form a square in the center. The maze is 40 centimeters from the floor. Mice were positioned in the center to explore for 5 min. The respective distances and times in the open and closed arms were recorded and analyzed using Etho Vision XT14 software.

### Y‐Maze

Spontaneous alternation of testing and training was performed as previously characterized.^[^ ^65^ ^]^ In short, the new arm was closed during the practice phase, and the mice were permitted to explore the starting arm and the old arm for only 10 min. After 1 h, trained mice were placed in the starting arm of the maze and the new arm was opened for exploration for 5 min.

### Three‐chamber Social Interaction Test

The social interaction experimental setup is a rectangular box that includes 3 separate chambers, each 25 × 325 cm, with adjacent chambers separated by a baffle with a door. The first phase was habituation, in which mice were positioned in the middle chamber and permitted to explore the 3 chambers for 10 min. The second stage was to place Stranger1 mice in a steel wire cage in one chamber, place an empty steel wire cage in the other chamber, and place the subject mice in the middle chamber for open exploration for 10 min. The third stage was to place Stranger2 mice in an empty steel wire cage in the other chamber, and the test mice were permitted to explore openly for 10 min. The trajectories of the test mice at each stage the time in each chamber, total active contact time around the steel wire cage were recorded and analyzed using Etho vision XT14 software.

### Rotarod Test

The experimental methodology was slightly modified as previously described.^[^ ^66^ ^]^ The experiment was conducted using a rotarod apparatus (Ugo Basile), the test began with training, which rapidly placed mice on a rod that gradually increased its speed from 4 to 40 rpm over 5 min, and the rotarod apparatus recorded the latency to fall, which was tested twice at 4‐h intervals within 1 day for a total of 3 days for each mouse.

### Grip Strength Test

Mouse grip strength measurements were made using the grip strength meter (BioSEB GS3) to gauge forelimb grip strength. The mice were grasped at the root of the tail in such a way that the front paws grabbed the grid attached to the force sensor and pulled smoothly backward until the grip force was released. Each experiment was repeated for 3 consecutive times and the test lasted for 3 days. The average value of 9 experiments was recorded.

### RNA‐Sequencing and Data Analysis

The mice embryo 13.5 days RNA from *Gsdmd^fl/fl^* and *Gsdmd^cko‐Nes^* cerebral cortex was extracted. The Agilent 2100 Bioanalyzer was used to control and quantify the quality of the RNA. Beijing Annoroad Corporation performed the RNA sequencing analysis using the Illumina HiSeq 2500 platform. All sequence data in this paper have been deposited in NCBI's GEO with accession number GSE 247 326.

### Statistical Analysis

All results were shown as mean ± SEM. All statistical analyses were performed using GraphPad Prism software 9.5. Statistical comparisons were analyzed using an unpaired two‐tailed Student's *t*‐test for 2 groups. For multiple comparisons, data were analyzed by 1‐way or 2‐way ANOVA. ^*^ *p* < 0.05, ^**^ *p* < 0.01, ^***^ *p* < 0.001, n.s., not significant.

## Conflict of Interest

The authors declare no conflict of interest.

## Author Contributions

H.M. and J.J. conceived the research. H.M. performed the experiments drafted the manuscript and analyzed the data. W.Z. did some plasmid construction. H.J. did some behavioral tests. J.Z., F.J., and W.W. provided some suggestions on experiments designed. C.Y. performed the mouse genotype identification experiment. J.J. supervised the project and acquired the funding support.

## Supporting information

Supporting Information

## Data Availability

The data that support the findings of this study are available in the supplementary material of this article.
