# Supplementary material for: Gasdermin D Mediated Mitochondrial Metabolism Orchestrate Neurogenesis Through LDHA During Embryonic Development
Source: Adv Sci (Weinh). 2024 Jul 21;11(35):2402285. doi: 10.1002/advs.202402285 (PMC11425199; doi:10.1002/advs.202402285)
Supplement: Supplementary file 1 — Supporting Information [file ADVS-11-2402285-s001.docx]

**Supporting Information for**

**Gasdermin D mediated mitochondrial metabolism orchestrate neurogenesis through LDHA during embryonic development**

Hongyan Ma,^1,2,3^ Huiyang Jia,^1,2,3^ Wenzheng Zou,^1,2,3^ FenJi,^1,2,3^ Wenwen Wang,^1,2,3^ Jinyue Zhao,^1,2,3^Chenqi Yuan, ^1,2,3^Jianwei Jiao^1,2,3,4,*^

Address for correspondence:

Jianwei Jiao

Email: jwjiao@ioz.ac.cn

**This PDF file includes:**

Supporting Information Figure legends

Supporting Information Figures S1 to S9

Supplementary Table S1-S3

**
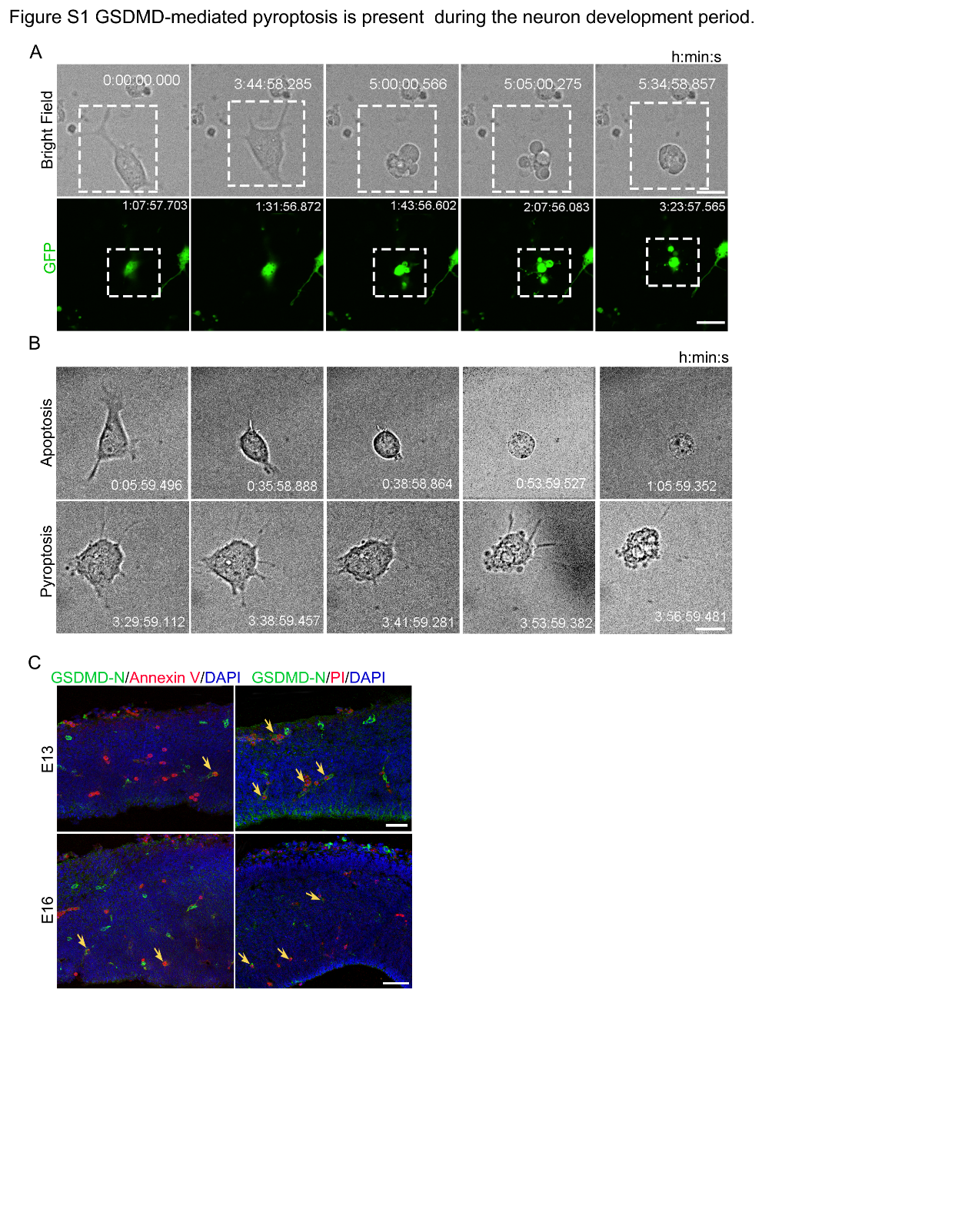
**

**Figure S1. Growth factors and LPS induce pyroptosis in mice and human neural progenitor cells.**

(**A**) Microscopy images of neural progenitor cells during pyroptosis. Shown are representative time-lapse cell images. Bright field direct observation of individual neuronal cells and GFP were observed by IUE the GFP plasmid to E13 fetal mice, isolating the GFP-positive cells 24 hours later. Scale bar,20μm.

(**B**) Microscopy images of human NPCs during apoptosis and pyroptosis. Shown are representative brightfield time-lapse cell images. Scale bar,20μm.

(**C**) Images of brain sections of the developing cerebral cortex ( E13 and E15 labeled forAnnexin and GSDMD-N.Propidium iodide (PI) was injected into the ventricles for 1 hours and the harvested brain sections were immunostained with GSDMD-N. Scale bar,E13=20μm, E13=50μm.

**
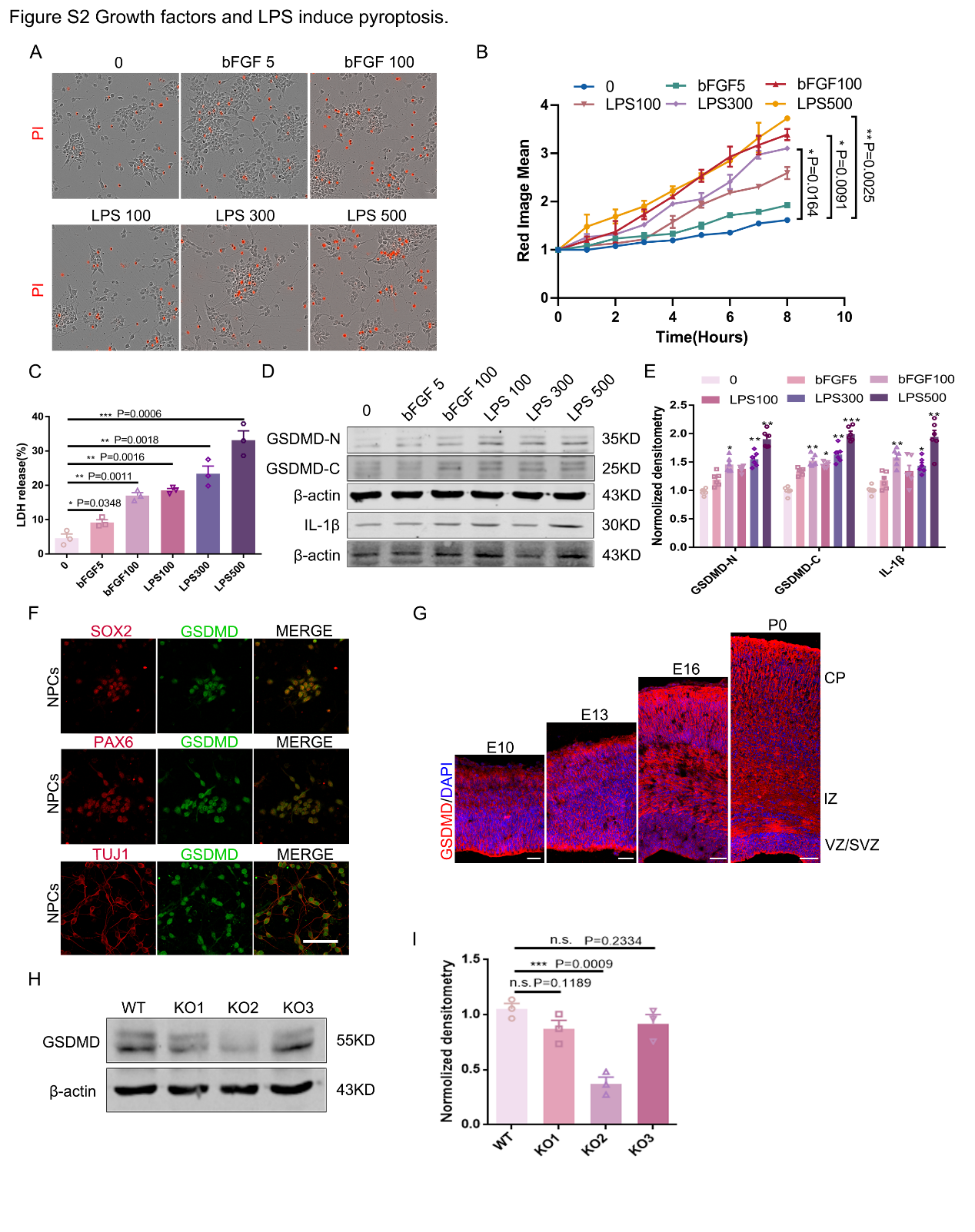
**

**Figure S2. GSDMD expression in the cerebral cortex.**

(**A**) Microscopy images showing PI uptake images of NPCs after treatment with bFGF(5, 50 ng mL^−1^) and LPS(1 ug/mL) of various concentrations for 16 h or 6h in proliferation medium, respectively. Scale bar,20μm.

(**B**) Quantification of the number of PI^+^ cells relative to time.

(**C**) Lactate dehydrogenase (LDH) release was measured in supernatant derived from primary neural progenitor cells treated as described. n = 3 independent experiments.

(**D**) Western blot analysis of GSDMD-N, GSDMD-C, and IL-1β, in NPCs after treatment with bFGF and LPS of various concentrations in proliferation medium.

(**E**)Quantification of the GSDMD-N, GSDMD-C and IL-1β protein levels. n= 6 independent experiments.

(**F**) GSDMD is co-labeled with Sox2 or Pax6 or Tuj1 in neural progenitor cells and neurons cultured in vitro. GSDMD is co-labeled with Sox2 or Pax6 or Tuj1 in neural progenitor cells and neurons cultured in vitro.

(**G**) Images of brain sections of the developing cerebral cortex ((E10, E13, E16, P0) labeled for GSDMD and DAPI. SVZ, subventricular zone; VZ, ventricular zone; IZ, intermediate zone; CP, cortical plate. Scale bar: E10, 20μm E13, 40μm, E16, 50μm, P0, 100μm.

(**H**)Western blot analysis western analyzed the knockdown efficiency of GSDMD knockdown human ES cell.

(**I**)The bar graph shows the normalized densitometry of GSDMD. n = 3 independent experiments.

Error bars represent means ± SEMs; 2-tailed unpaired t-test; one-way ANOVA with Dunnett’s multiple-comparison correction. *p < 0.05, **p < 0.01, ***p < 0.001; n.s., not significant.

**
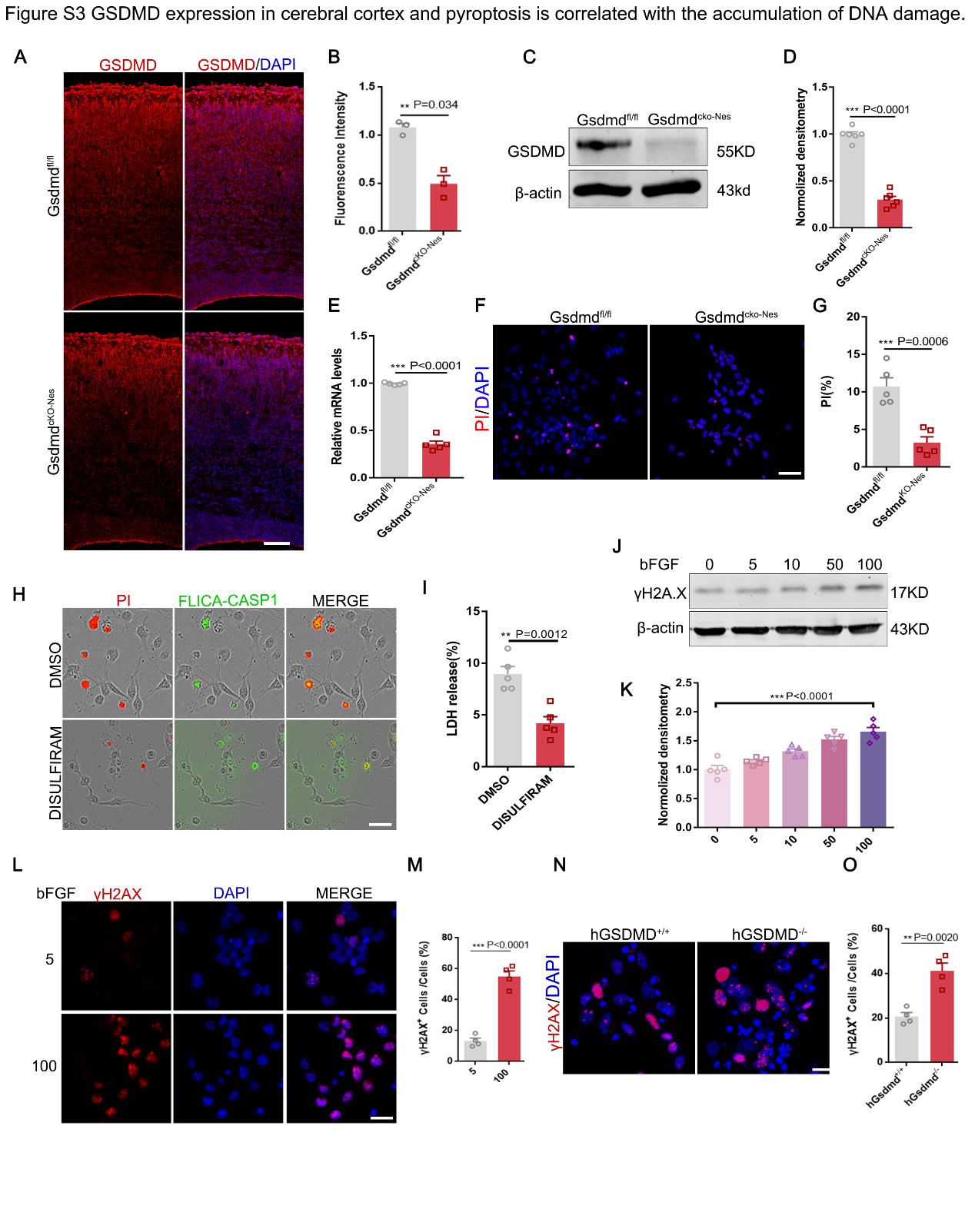
**

(**A**) Immunostaining shows that the expression of GSDMD is reduced in E13 GSDMD cKO brains.

(**B**) Quantification of the GSDMD fluorescence intensity in *Gsdmd^fl/fl^* mice and *Gsdmd^cKO-Nes^* mice. n = 3 independent experiments.

(**C**)Western blot analysis showed that the GSDMD expression was reduced in the NPCs of *Gsdmd*^cKO-Nes^ mice.

(**D**)The bar graph shows the normalized densitometry of GSDMD. n = 6 independent experiments.

(**E**) RT-PCR was performed to detect the mRNA levels of GSDMD at E13 cortex. n = 6 independent experiments.

(**F**) Representative micrographs of PI and DAPI staining of NPCs isolated from *Gsdmd^fl/fl^* and *Gsdmd^cko/Nes^* mice. Scale bar,100 μm.

(**G**) Quantification of the percentage of PI^+^ cells is shown in the histogram. n =5 independent experiments.

(**H**) NPCs were isolated from *Gsdmd^fl/fl^*mice and treated with DMSO or Disulfiram for 12 h, and cells were stained with FLICA-CASP1 for 1 h and Propidium iodide. The images were visualized using microscopy. Scale bar, 50μm.

(**I**) LDH release was measured of supernatants *Gsdmd^fl/fl^* mice and treated with DMSO or Disulfiram for 12 h. n =5 independent experiments.

(**J**)Western blot analysis of 𝛾H2AX in NPCs after treatment with bFGF (5, 50 ng mL^−1^) of various concentrations for 16 h in proliferation medium.

(**K**)The bar graph showed 𝛾H2AX normalized densitometry. n = 5 independent experiments.

(**L**)NPCs were isolated at E13 and then treated with bFGF (5, 50 ng mL^−1^) for 16 h in proliferation medium. Then cells were fixed and stained with anti-𝛾H2AX antibodies. Scale bar, 20 µm.

(**M**)The bar graph shows the fluorescence intensity of 𝛾H2AX (n = 6 field of view of three individual experiments).

(**N**)Confocal immunofluorescence image of 𝛾H2AX in *GSDMD^+/+^* and *GSDMD^+/+^*human NPCs. Scale bar,20μm.

(**O**)Quantification of the proportion of 𝛾H2AX^+^ and GFP^+^ among GFP^+^ cells in human NPCs with control and h*GSDMD*-shRNA lentivirus-infected. n = 5 independent experiments.

**
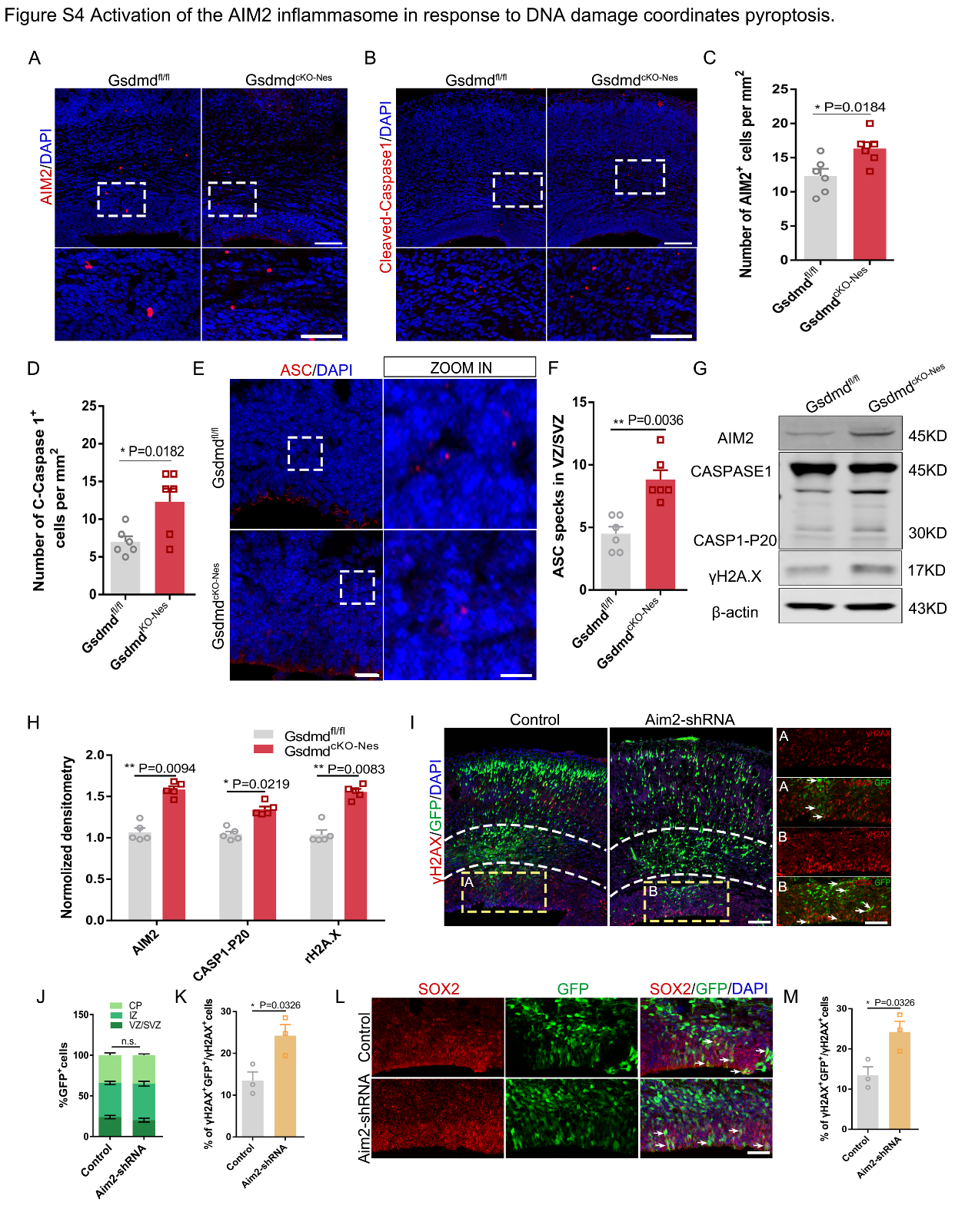
**

**Figure S4. Activation of the AIM2 inflammasome in response to DNA damage coordinates pyroptosis.**

(**A**)Representative confocal images of AIM2 staining at E16 cerebral cortex. Scale bar,100μm. The next is higher magnification images. Scale bar,50μm.

(**B**)Representative confocal images of Cleaved-Caspase-1 at E16 cerebral cortex. Scale bar,100μm. The next is higher magnification images. Scale bar,50μm.

(**C**)Quantification showing the increased number of AIM2^+^cells in *Gsdmd^cKO-Nes^* mice. n = 6 independent experiments.

(**D**)Quantification showing the increased number of Cleaved-Caspase1^+^cells in *Gsdmd^cKO-Nes^* mice. n = 6independent experiments.

(**E**) Representative confocal images of ASC specks at E16 cerebral cortex. Scale bar,50μm. The right is higher magnification images. Scale bar,100μm.

(**F**)Quantification showing the increased number of ASC specks in *Gsdmd^cKO-Nes^* mice.

(**G**)Western blot analysis of AIM2, Caspase-1, Caspase-1-p20, 𝛾H2AX protein levels in E16 *Gsdmd ^fl/fl^*mice and *Gsdmd ^cKO-Nes^* mice brain.

(**H**)The bar graph shows the normalized densitometry of AIM2, Caspase1, Caspase1-p20, 𝛾H2AX. n = 5 independent experiments.

(**I**) The control and Aim2-shRNA plasmid was electroporated into the E13 mouse brains, and the mice were sacrificed at E16. γH2AX staining at E16 cerebral cortex, White arrows indicate cells co-labeled with γH2AX and green fluorescence. Scale bar,20μm.

(**J**) Graphs of the percentage of GFP-positive cells in the VZ/SVZ, IZ, and CP. n=3 independent experiments.

(**K**) Percentage of γH2AX ^+^GFP^+^ cells in control or Aim2-shRNA-electroporated brains. n = 3 independent experiments.

(**L**)Representative images of E16 cortices electroporated with Control, Aim2-shRNA, and immunostained for SOX2. Scale bar,50 μm. White arrows indicate cells co-labeled with SOX2 and GFP-positive cells. Scale bar,20μm.

(**M**) Percentage of SOX2^+^GFP^+^ cells in control or Aim2-shRNA-electroporated brains. n = 3 independent experiments.

Error bars represent means ± SEMs; 2-tailed unpaired t-test; one-way ANOVA with Dunnett’s multiple-comparison correction. *p < 0.05, **p < 0.01, ***p < 0.001; n.s., not significant.

**
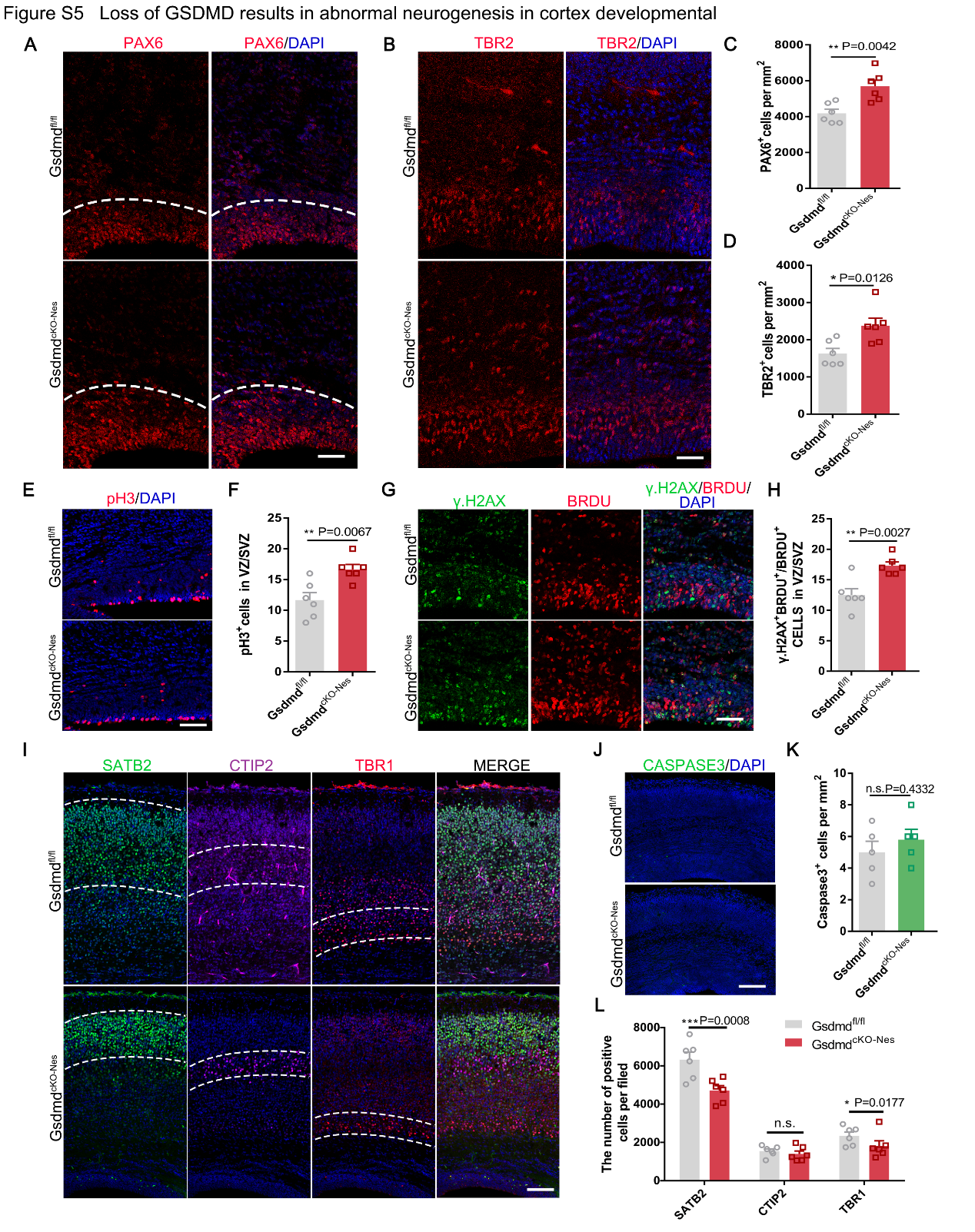
**

**Figure S5. Loss of GSDMD results in abnormal neurogenesis in early developmental window.**

(**A**)Brain sections of *Gsdmd^fl/fl^* mice and *Gsdmd^cKO-Nes^* mice at E16 were immunostained with PAX6 and DAPI. Scale bar,100μm.

(**B**)Brain sections of *Gsdmd^fl/fl^* mice and *Gsdmd^cKO-Nes^* mice at E16 were immunostained with TBR2 and DAPI. Scale bar,100μm.

(**C**)Quantitative analyses of the PAX6^+^ cells at E16 cerebral cortex. n = 6 independent experiments.

(**D**)Quantitative analyses of the TBR2^+^ cells at E16 cerebral cortex. n = 6 independent experiments.

(**E**)Immunostaining for the mitotic marker pH3 in *Gsdmd^fl/fl^* mice and *Gsdmd^cKO-Nes^* mice cortices at E16. Scale bars, 20 μm.

(**F**)Quantification of pH3^+^ mitotic cells in the ventricular zone and subventricular zone (VZ/SVZ). n = 6 independent experiments.

(**G**)Representative images of E16 coronal brain sections were immunostained for 𝛾H2A.X and BrdU. BrdU was injected intraperitoneally into pregnant mice at E16 for 2 hours of pulse labeling. Scale bars, 50μm.

(**H**)Percentage of 𝛾H2A.X^+^BrdU^+^ cells among all BrdU^+^ cells in VZ/SVZ. n = 6 independent experiments.

(**I**)Representative images of SATB2, CTIP2, and TBR1 staining at P0 cerebral cortex. Scale bars, 200 μm.

(**J**)E16 neocortex sections of *Gsdmd^fl/fl^* mice and *Gsdmd^cKO-Nes^* mice were immunostained with Caspase-3 and DAPI. Scale bar, 100 μm.

(**K**)The graph shows the numbers of Caspase3^+^ cells per field. n = 5 independent experiments.

(**L**)The bar graph shows the number of SATB2^+^, CTIP2^+^and TBR1^+^ cells per 200000 µm^2^. n = 6 independent experiments.

Error bars represent means ± SEMs; 2-tailed unpaired t-test; one-way ANOVA with Dunnett’s multiple-comparison correction. *p < 0.05, **p < 0.01, ***p < 0.001; n.s., not significant.

**
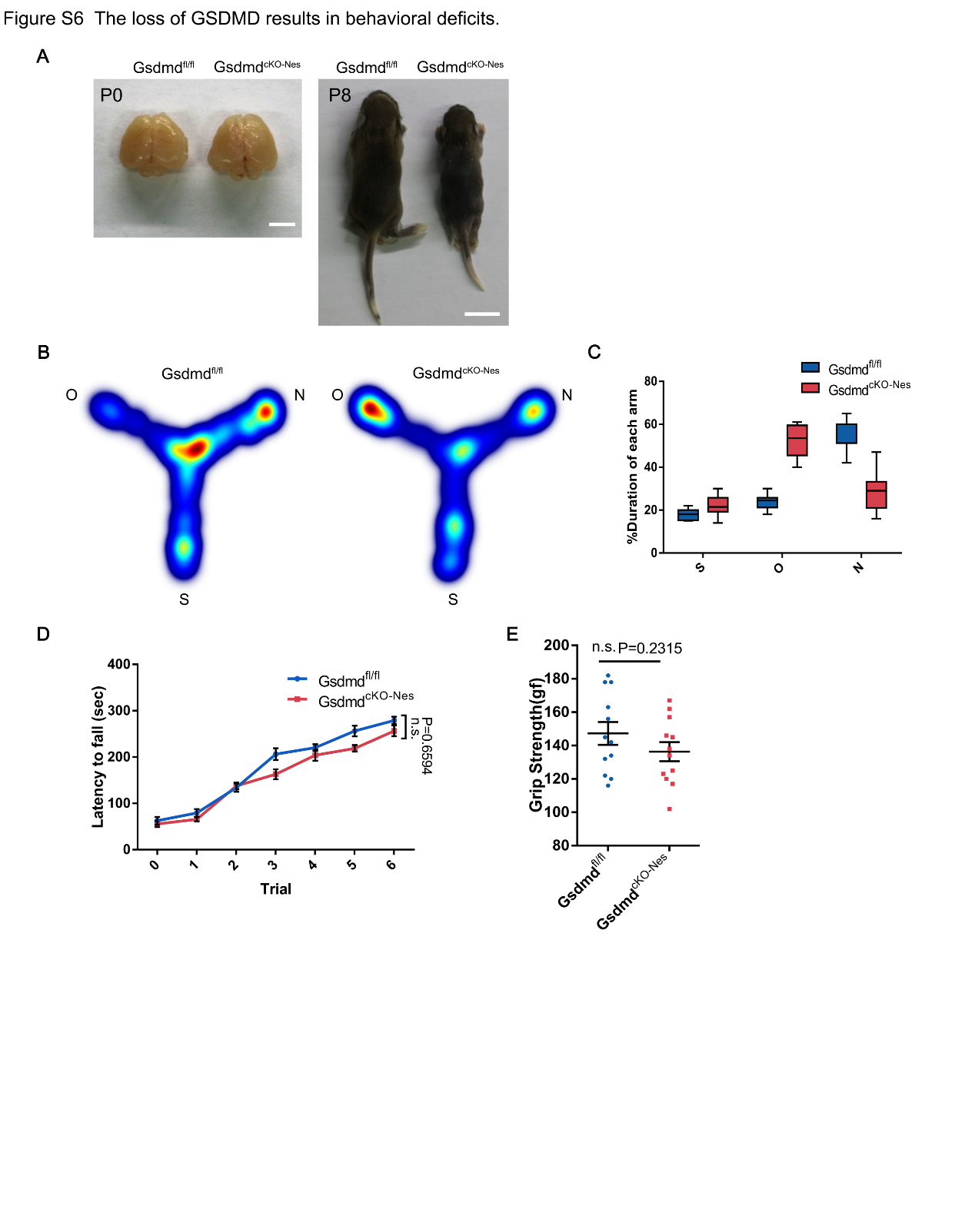
**

**Figure S6. The loss of GSDMD results in behavioral deficits.**

(**A**) The left representative images of the *Gsdmd^fl/fl^* mice and *Gsdmd^cKO-Nes^* mice brain sizes at P0. Scale bar, 10 mm. The right representative images of the *Gsdmd^fl/fl^* mice and *Gsdmd^cKO-Nes^* mice body sizes at P8. Scale bar, 1 mm.

(**B**) Representative tracks in the Y-maze test. S: start;O: old;N:new.

(**C**) Compared with *Gsdmd^fl/fl^* mice and *Gsdmd^cKO-Nes^* mice spent less time in the new arms and more time in the old arms. n=12 *Gsdmd^fl/fl^* and *Gsdmd^cKO-Nes^* mice.

(**D**) The rotarod test shows that the forced locomotor activity was no significant difference between *Gsdmd^fl/fl^* mice and *Gsdmd^cKO-Nes^* mice. n=12 *Gsdmd^fl/fl^* mice and *Gsdmd^cKO-Nes^* mice.

(**E**) The grip strength test shows the force of grip strength was no significant difference between *Gsdmd^fl/fl^* mice and *Gsdmd^cKO-Nes^* mice. n=12 *Gsdmd^fl/fl^* and *Gsdmd^cKO-Nes^* mice.

Error bars represent means ± SEMs; 2-tailed unpaired t test; one-way ANOVA with Dunnett’s multiple-comparison correction. *p < 0.05, **p < 0.01, ***p < 0.001; n.s., not significant.

**
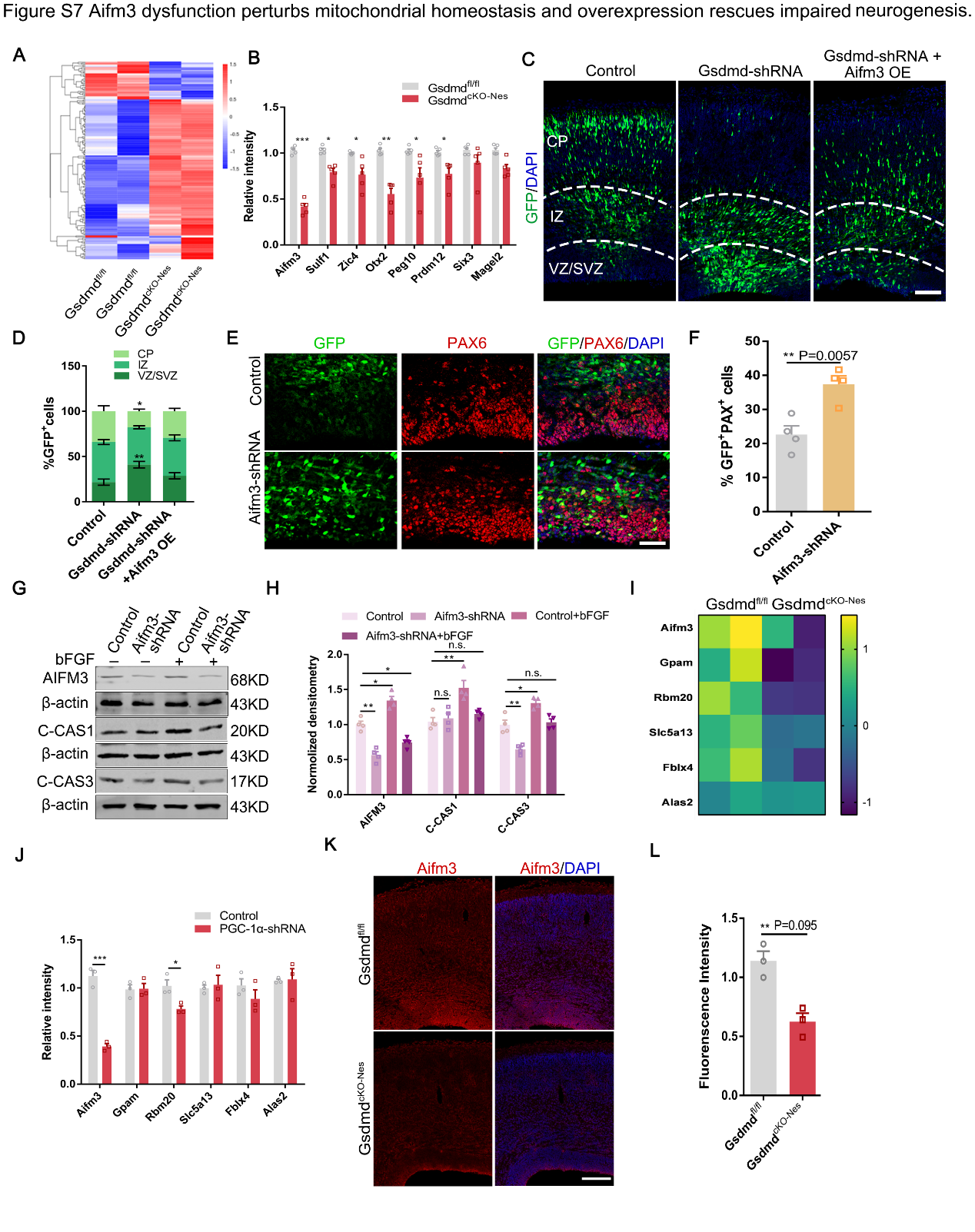
**

**Figure S7. Aifm3 dysfunction perturbs mitochondrial homeostasis and overexpression rescues impaired neurogenesis.**

(**A**) Heatmap shows global genes were sorted by fold change.

(**B**)Eight significantly expressed genes were analyzed by qPCR in NPCs of *Gsdmd^fl/fl^* and *Gsdmd^cKO-Nes^*, of which Aifm3 expression has decreased. n = 5 independent experiments.

(**C**)GSDMD knockdown caused abnormal GFP-positive cell distribution in the embryonic brain, and the Aifm3 overexpression could rescue this phenotype partially.

(**D**)Graphs of the percentage of GFP-positive cells in the VZ/SVZ, IZ, and CP. n=4 independent experiments.

(**E**)Representative images of E16 cortices electroporated with Control, Aifm3-shRNA, and immunostained for PAX6 and DAPI. Scale bar,50 μm.

(**F**)The bar graph shows the percentage of GFP^+^ PAX6^+^ cells. n=4 independent experiments.

(**G**) Western blot analysis of AIFM3, C-CAS1, and C-CAS3 protein levels in control and Aifm3-shRNA lentivirus-infected NPCs, and then treated with bFGF (50, 500 ng mL^−1^) for 24 h in proliferation medium, respectively.

(**H**) The bar graph shows the normalized densitometry of AIFM3, C-CAS1, and C-CAS3. n = 4 independent experiments.

(**I**) A heat map of six mitochondria-associated localized differentially expressed genes from RNA-Seq in E13 brain was further analyzed. the Aifm3 gene was one of the significantly differentially expressed genes.

(**J**)RT-PCR analysis of the mitochondria-associated genes expression levels in control and PGC-1α-shRNA lentivirus-infected NPCs. n = 3 independent experiments.

(**K**) Immunostaining shows that the expression of Aifm3 is reduced in E13 GSDMD cKO brains.

(**L**) Quantification of the Aifm3 expression intensity in *Gsdmd^fl/fl^* mice and *Gsdmd^cKO-Nes^* mice. n = 3 independent experiments.

Error bars represent means ± SEMs; 2-tailed unpaired t-test; one-way ANOVA with Dunnett’s multiple-comparison correction. *p < 0.05, **p < 0.01, ***p < 0.001; n.s., not significant.

**
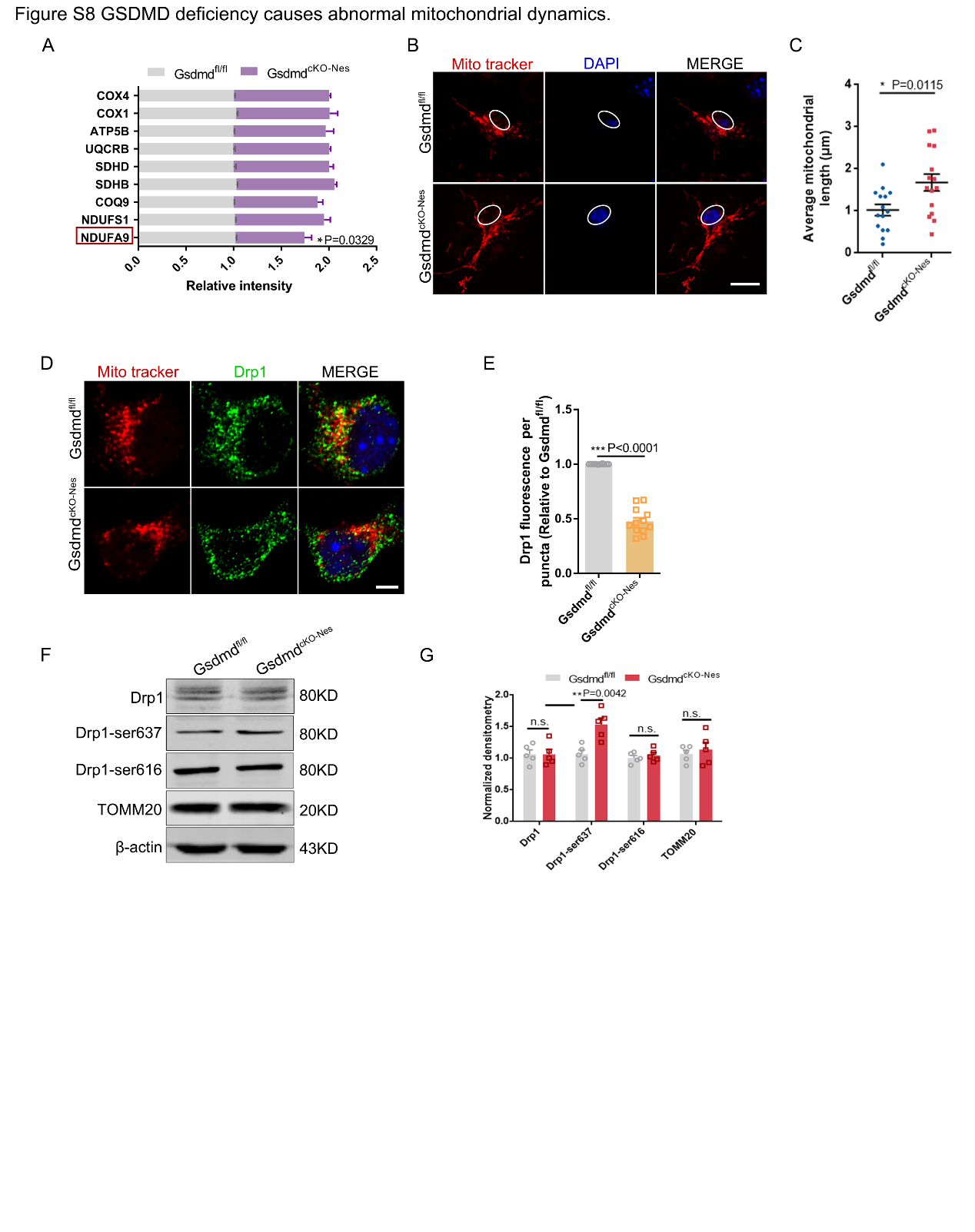
**

**Figure S8. Gasdermin D deficiency regulates mitochondrial dynamics in NPCs.**

(**A**)RT-PCR analysis of the representative ETC complex genes. The statistical significance between *Gsdmd^fl/fl^* and *Gsdmd^cKO-Nes^* is shown at the bottom. The most significantly down-regulated gene, Ndufa9, is indicated by the red box in the figure. n = 5 independent experiments.

(**B**)Confocal immunofluorescence image of Mito tracker labeling in *Gsdmd^fl/fl^* and *Gsdmd^cKO-Nes^* NPCs. Scale bars, 2 μm.

(**C**)Quantitative analysis showed the length per mitochondrial in *Gsdmd^fl/fl^* and *Gsdmd^cKO-Nes^* NPCs. n = 15 from three independent biological replicates.

(**D**)Confocal immunofluorescence image of Mito tracker and DRP1 in *Gsdmd^fl/fl^* and *Gsdmd^cKO-Nes^* NPCs showing the subcellular localization. Scale bar, 10 μm.

(**E**)The bar graph shows the Drp1 fluorescence intensity per puncta. The data are normalized to the *Gsdmd^fl/fl^*. n = 12 from three independent experiments.

(**F**)Western blot analysis of Drp1, Drp1-Ser637, Drp1-Ser616 and TOMM20 protein levels in *Gsdmd^fl/fl^* mice and *Gsdmd^cKO-Nes^* mice.

(**G**)The bar graph shows the normalized densitometry of Drp1, Drp1-Ser637, Drp1-Ser616 and TOMM20. n = 5 independent experiments.

Error bars represent means ± SEMs; 2-tailed unpaired t test; one-way ANOVA with Dunnett’s multiple-comparison correction. *p < 0.05, **p < 0.01, ***p < 0.001; n.s., not significant.


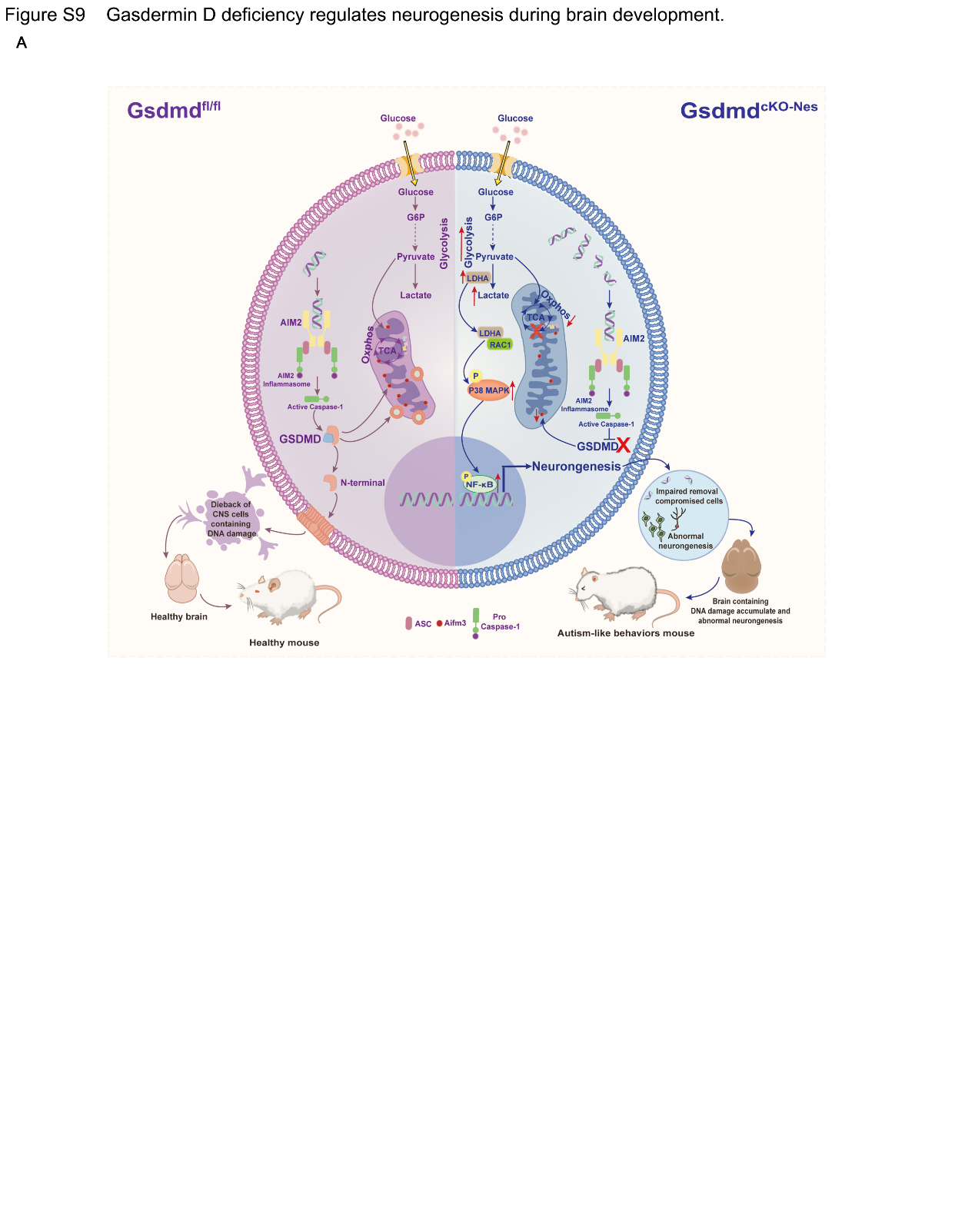


**Figure S9. Gasdermin D deficiency regulates neurogenesis during brain development.**

Schematic illustration showing the role of GSDMD in the activation of immune activation in neurodevelopment under DNA damage-activated AIM2 inflammation. Deletion of GSDMD not only leads to defective immune activation as well as impaired mitochondrial dynamics but also affects cellular metabolism by regulating downstream molecules Aifm3, all of this leads to aberrant neurogenesis and autism-like behaviors in mice.

Error bars represent means ± SEMs; 2-tailed unpaired t test; one-way ANOVA with Dunnett’s multiple-comparison correction. *p < 0.05, **p < 0.01, ***p < 0.001; n.s., not significant.

**Table S1: RT-PCR Primers**

| **Name** | **Sequence (5’-3’)** |
| --- | --- |
| GSDMD-RT-F | ATGCCATCGGCCTTTGAGAAA |
| GSDMD-RT-R | AGGCTGTCCACCGGAATGA |
| AIFM3-RT-F | TAGAGCTGGAGGAGACACCC |
| AIFM3-RT-R | CGCAACTCTGACACCTCTGT |
| ZIC4-RT-F | ACCGCTTCTCATGCACGTTA |
| ZIC4-RT-R | AAAAGGTTCCGACCTCGCAT |
| SULF1-RT-F | CGGGTAACAGGTTTCGGACA |
| SULF1-RT-R | TGGGCAAGTGGTTTGACTGT |
| OTX2-RT-F | TGAGGCCTGCCAAGAAGAAG |
| OTX2-RT-R | GACAAGGGGTCAGACAGTGG |
| PEG10-RT-F | TGCTTGCACAGAGCTACAGTC |
| PEG10-RT-R | AGTTTGGGATAGGGGCTGCT |
| PRDM12-RT-F | CAGCCCAAAAACGGCCTTC |
| PRDM12-RT-R | TGGAACTCTGAGCGATAATCACT |
| SIX3-RT-F | CCGGAAGAGTTGTCCATGTTC |
| SIX3-RT-R | ACTCGTGTTTGTTGATGGCCT |
| MAGEL2-RT-F | AATGCCGCATGTTCCCATTAC |
| MAGEL2-RT-R | ATGGATCATCACGACACCAGG |
| ALDOA-RT-F | CGTGTGAATCCCTGCATTGG |
| ALDOA-RT-R | CAGCCCCTGGGTAGTTGTC |
| GAPDH-RT-F | AGGTCGGTGTGAACGGATTTG |
| GAPDH-RT-R | TGTAGACCATGTAGTTGAGGTCA |
| HK2-RT-F | GTGTGCTCCGAGTAAGGGTG |
| HK2-RT-R | CAGGCATTCGGCAATGTGG |
| PGK1-RT-F | ATGTCGCTTTCCAACAAGCTG |
| PGK1-RT-R | GCTCCATTGTCCAAGCAGAAT |
| LDHA-RT-F | TGTCTCCAGCAAAGACTACTGT |
| LDHA-RT-R | GACTGTACTTGACAATGTTGGGA |
| LDHB-RT-F | CATTGCGTCCGTTGCAGATG |
| LDHB-RT-R | GGAGGAACAAGCTCCCGTG |
| NDUFA9-RT-F | GTCCGCTTTCGGGTTGTTAGA |
| NDUFA9-RT-R | CCTCCTTTCCCGTGAGGTA |
| NDUFS1-RT-F | TGCAAATCCCTCGATTCTGTTAC |
| NDUFS1-RT-R | GCTTTCTCAATCTCTACCAGGC |
| COQ9-RT-F | GTGGGGTTCCGGTCTTCAG |
| COQ9-RT-R | GGGGTGGACGGGAAAACTC |
| SDHB-RT-F | ATTTACCGATGGGACCCAGAC |
| SDHB-RT-R | GTCCGCACTTATTCAGATCCAC |
| SDHD-RT-F | TGGTCAGACCCGCTTATGTG |
| SDHD-RT-R | GGTCCAGTGGAGAGATGCAG |
| UQCRB-RT-F | GGCCGATCTGCTGTTTCAG |
| UQCRB-RT-R | CATCTCGCATTAACCCCAGTT |
| COX1-RT-F | ATGAGTCGAAGGAGTCTCTCG |
| COX1-RT-R | GCACGGATAGTAACAACAGGGA |
| COX4-RT-F | TCCCCACTTACGCTGATCG |
| COX4-RT-R | GATGCGGTACAACTGAACTTTCT |
| ATP5B-RT-F | TCCTGCCAGAGACTATGCG |
| ATP5B-RT-R | GATGACTGCCACGATTCGC |
| β-actin-RT-F | GGCTGTATTCCCCTCCATCG |
| β-actin-RT-R | CCAGTTGGTAACAATGCCATGT |

**Table S2: Primer sequences target AIFM3**

| **Name** | **Sequence (5’-3’)** |
| --- | --- |
| AIFM3-2K-F | ACAGATCGACCGTCCGTCG |
| AIFM3-2K-R | GTCCCCAGGTCAGGACGTC |
| AIFM3-1K-F | CTGGAGCTCTTACCGGTCTA |
| AIFM3-1K-R | CCCTGCACACGCTTCCTTTCG |
| AIFM3-0.5K-F | TCCTCATGACCCTGACACGCG |
| AIFM3-0.5K-R | CCGTTCGTTCGTCCGGGATCC |
| AIFM3-CDS-F | AACACAAGTTCCTACCGAAGT |
| AIFM3-CDS-R | GATCGACCGATGCCTCTTCCG |

**Table S3:KEY RESOURCES TABLE**

| **REAGENT or RESOURCE** | **SOURCE** | **IDENTIFIER** |
| --- | --- | --- |
| **Antibodies** | | |
| Rabbit Anti-GSDMD | Proteintech | Cat#20770-1-AP; RRID:AB_10696319 |
| Rabbit Anti-GSDMD | Abcam | Cat#ab209845; RRID: AB_2783550 |
| Rabbit Anti-Cleaved  Gasdermin D (Asp276) | Cell Signaling Technology | Cat#36425S; RRID: AB_2799099 |
| Rabbit Anti-Caspase 1/p20/p10 | Proteintech | Cat#22915-1-AP; RRID:AB_ 2876874 |
| Rabbit Anti-Cleaved-Casp1  -p20 (Asn120) | Solarbio | Cat#22915-1-AP; RRID: AB_2876874 |
| Rabbit Anti-AIFM3 | Proteintech | Cat#14778-1-AP; RRID: AB_2258090 |
| Mouse Anti-IL-1β | R&D Systems | Cat#AF-401-NA; RRID: AB_416684 |
| Rabbit Anti-LDHA | Proteintech | Cat#19987-1-AP; RRID: AB_10646429 |
| Rat Anti-BrdU | Abcam | Cat#ab6326; RRID: AB_305426 |
| Rabbit Anti-γ.H2AX | Cell Signaling Technology | Cat# 9718S; RRID: AB_2118009 |
| Mouse Anti-Tuj1) | Millipore | Cat#MAB1637; RRID: AB_2210524 |
| Rabbit Anti-PAX6 | Abcam | Cat#ab5790; RRID: AB_305110 |
| Rabbit anti-SOX2 | Cell Signaling Technology | Cat#3728; RRID: AB_2194037 |
| Rabbit Anti-Ki67 | Abcam | Cat#ab15580; RRID: AB_443209 |
| Rabbit Anti-pH3 | Cell Signaling Technology | Cat#3377S; RRID: AB_1549592 |
| Rabbit anti-Cleaved caspase3 | Cell Signaling Technology | Cat# 9664S; RRID: AB_2070042 |
| Rabbit Anti-TBR2 | Abcam | Cat#ab23345; RRID: AB_778267 |
| Rat Anti-Ctip2 | Abcam | Cat#ab18465; RRID: AB_2064130 |
| Mouse Anti-SATB2 | Abcam | Cat#ab51502; RRID: AB_882455 |
| Rabbit Anti-TBR1 | Abcam | Cat#ab31940; RRID: AB_2200219 |
| Rabbit Anti-DRP1 | Abclonal | Cat# A2586; RRID: AB_2764472 |
| Rabbit Anti-P-DRP1 (Ser637) | Beyotime Biotechnology | Cat# AF5791; |
| Mouse Anti-Tom20 | Santa Cruz Biotechnology | Cat# sc-17764; RRID: AB_628381 |
| Mouse Anti-GSDMD-N | Affinity | Cat# DF13758; RRID: AB_3076218 |
| Rabbit Anti-P-DRP1 (Ser616) | Affinity | Cat# DF2972; RRID: AB_2840952 |
| Rabbit Anti-Annexin V | Proteintech | Cat# 11060-1-AP; RRID: AB_2057585 |
| Rabbit Anti-TOMM20 | Beyotime Biotechnology | Cat# AF1717; |
| Mouse Anti-FLAG | Sigmal | Cat# F1804; RRID: AB_262044 |
| Rabbit Anti-HA | Abmart | Cat#M20003; RRID: AB_2864345 |
| Rabbit Anti PGC-1αRabbit mAb | Cell Signaling Technology | Cat#2178; RRID: AB_823600 |
| Rabbit Anti SirT1 | Cell Signaling Technology | Cat#2028; RRID: AB_1196631 |
| Rabbit Anti AMPK-alpha | Cell Signaling Technology | Cat#2532; RRID: AB_330331 |
| Rabbit Anti- pNF-κB p65 | Cell Signaling Technology | Cat# 3033T; RRID: AB_331284 |
| Rabbit Anti-NF-κB p65 | Cell Signaling Technology | Cat# 6956S; RRID: AB_10828935 |
| Rabbit Anti-p-P38 MAPK | Cell Signaling Technology | Cat# 4511; RRID: AB_2139682 |
| Rabbit Anti- P38 MAPK | Cell Signaling Technology | Cat# 8690S; RRID: AB_10999090 |
| Rabbit Anti-β-ACTIN | Proteintech | Cat#20536-1-AP; RRID: AB_10700003 |
| Mouse anti-β- ACTIN | Proteintech | Cat# 60008-1-Ig; RRID: AB_2289225 |
| **Chemicals, peptides** | | |
| Papain | Worthington | Cat# 38P18865 |
| TRIzol | Ambion Life Technolog | Cat# 15596018 |
| MitoTracker | ThermoFisher Scientific | Cat# M22426 |
| Anti-HA-tag magnetic beads | MBL | Cat# M132-11 |
| Anti-FLAG-tag magnetic beads | MBL | Cat# M185-11 |
| Anti-IgG beads | Invitrogen | Cat# 10004D |
| DAPI | ThermoFisher Scientific | Cat# D35 |
| Propidium iodide | Sigma-Aldrich | Cat# P4170 |
| Lipopolysaccharide | Sigma-Aldrich | Cat# L4391 |
| Belnacasan (VX-765) | selleck | Cat# S2228 |
| Disulfiram | Sigma-Aldrich | Cat# PHR1690-1G |
| Adezmapimod | Selleck | Cat# SB203580 |
| Co-IP Lysis buffer | Beyotime Biotechnology | Cat# P0013 |
| RIPA Lysis buffer | Solarbio | Cat# R0010 |
| PBS | Beyotime Biotechnology | Cat# C0221A |
| DPBS | GIBCO | Cat# C14190500CP |
| Dulbecco’s Modified Eagle  Medium (DMEM) | GIBCO | Cat# 11995-065 |
| Low Glucose DMEM | GIBCO | Cat# 11885-084 |
| DMEMF/12 medium | GIBCO | Cat# 11330-032 |
| Neurobasal medium | GIBCO | Cat# 21103-049 |
| Fetal Bovine Serum (FBS) | GIBCO | Cat# 16000044 |
| Penicillin / Streptomycin | Invitrogen | Cat# 15070063 |
| Poly-D-Lysine | Sigma-Aldrich | Cat# P3655 |
| Laminin | Invitrogen | Cat# 23017015 |
| B27 supplement without VA | Invitrogen | Cat# 12587010 |
| B27 supplement with VA | Invitrogen | Cat# 17504-044 |
| 100 × Gluta MAX | Invitrogen | Cat# 35050061 |
| MEM Non-Essential Amino  Acids Solution | GIBCO | Cat# 11140050 |
| EGF | Invitrogen | Cat# PHG0311 |
| bFGF | Invitrogen | Cat# PHG0026 |
| DMEM essential 8 medium | GIBCO | Cat# A1517001 |
| 100 × N2 supplement | Thermo Fisher | Cat# 17502048 |
| Dorsomorphin | Selleck | Cat# S7840 |
| SB431542 | Tocris | Cat# 1614 |
| CHIR99021 | Stemgent | Cat# 04-0004-10 |
| Compound E | EMD Chemicals | Cat# 209986-17-4 |
| human LIF | Millipore | Cat# LIF1010 |
| Accutase | Thermo Fisher | Cat# A1110501 |
| Matrigel hESC | CORNING | Cat# 354277 |
| **Critical commercial assays** | | |
| FAM-FLICA® Caspase-1 (YVAD) Assay Kit | ImmunoChemistry Technologies | Cat# 97 |
| Fast Quant RT Kit | TIANGEN | Cat# KR106-02 |
| SuperReal PreMix Plus (SYBR Green) PCR Kit | TIANGEN | Cat# FP205-02 |
| L-LA Assay Kit | Solarbio | Cat# BC2235 |
| CytoTox 96 Non-Radioactive Cytotoxicity Assay kit | Promega G1780 | Cat# G1780 |
| Potential Assay Kit with JC-1 | Beyotime Biotechnology | Cat# C2006 |
| **Deposited data** | | |
| Raw and analyzed data of bulk  RNA-seq | This paper | GSE 247326 |
| **Experimental models: Cell lines** | | |
| HEK293 Cell Line | ATCC | CRL-1573; RRID: CVCL_0045 |
| Mouse neuroblastoma N2a | ATCC | CCL-131; RRID: CVCL_0470 |
| H9 hESCs | WiCell CVCL_9773) | RRID: CVCL_9773 |
| Experimental Models: Organisms/Strains | | |
| C57BL/6 | Jackson Laboratory | Stock No: 000664;  RRID: IMSR_JAX:000664 |
| Mouse: Nestin-cre | Jackson Laboratory | Stock No: 019103;  RRID: IMSR_JAX:019103 |
| Mouse: GSDMD^fl/fl^ | RIKEN BRC | RBRC 10762  RRID: IMSR_RBRC10762 |
| **Software and algorithms** | | |
| Zen Microscope software | Zeiss | https://www.zeiss.com/microscopy/int/  products/microscope-software/zen.html |
| LAX | Leica | <https://www.leica-microsystems>.com/ products/microscope-software/p/leica-las-x-ls/downloads/ |
| Imaris 9.7 | Imaris | http://www.bitplane.com/imaris/imaris |
| CaspLab-Comet Assay Software | CASP Lab | https://sourceforge.net/projects/casp/ |
| ImageJ | ImageJ Wiki | https://imagej.net/Downloads |
| ABI7500 real-time PCR system | Applied Biosystems | Applied Biosystems |
| Avisoft SASLab Pro | Avisoft Bioacoustics | https://www.avisoft.com/downloads/ |
| Avisoft RECORDER USGH | Avisoft Bioacoustics | https://www.avisoft.com/downloads/ |
| LI-COR Image Studio | LI-COR Biosciences | LI-COR Biosciences |
| Seahorse Wave | Agilent | <http://www.agilent.com/enus/products/>  cell-analysis-(seahorse)/software download-for-wave- |
| GraphPad Prism 9.5 | GraphPad | https://www.graphpad.com/ |
| IncuCyte S3 2018C | Sartorius | https://www.sartorius.com/en/products/live-cell-imaging-analysis/live-cell-analysis-software |
